# Exploring the Key Genes and Identification of Potential Diagnosis Biomarkers in Alzheimer’s Disease Using Bioinformatics Analysis

**DOI:** 10.3389/fnagi.2021.602781

**Published:** 2021-06-14

**Authors:** Wuhan Yu, Weihua Yu, Yan Yang, Yang Lü

**Affiliations:** ^1^Department of Geriatrics, The First Affiliated Hospital of Chongqing Medical University, Chongqing, China; ^2^Institutes of Neuroscience, Chongqing Medical University, Chongqing, China; ^3^State Key Laboratory of Power Transmission Equipment and System Security and New Technology, College of Electrical Engineering, Chongqing University, Chongqing, China

**Keywords:** Alzheimer’s disease, diagnosis biomarkers, hub genes, integrative analysis, aging

## Abstract

**Background:**

Alzheimer’s disease (AD) is one of the major threats of the twenty-first century and lacks available therapy. Identification of novel molecular markers for diagnosis and treatment of AD is urgently demanded, and genetic biomarkers show potential prospects.

**Method:**

We identify and intersected differentially expressed genes (DEGs) from five microarray datasets to detect consensus DEGs. Based on these DEGs, we conducted Gene Ontology (GO), performed the Kyoto Encyclopedia of Genes and Genomes (KEGG) enrichment analysis, constructed a protein—protein interaction (PPI) network, and utilized Cytoscape to identify hub genes. The least absolute shrinkage and selection operator (LASSO) logistic regression was applied to identify potential diagnostic biomarkers. Gene set enrichment analysis (GSEA) was performed to investigate the biological functions of the key genes.

**Result:**

We identified 608 consensus DEGs, several dysregulated pathways, and 18 hub genes. Sixteen hub genes dysregulated as AD progressed. The diagnostic model of 35 genes was constructed, which has a high area under the curve (AUC) value in both the validation dataset and combined dataset (AUC = 0.992 and AUC = 0.985, respectively). The model can also differentiate mild cognitive impairment and AD patients from controls in two blood datasets. Brain-derived neurotrophic factor (BDNF) and WW domain-containing transcription regulator protein 1 (WWTR1), which are associated with the Braak stage, Aβ 42 levels, and β-secretase activity, were identified as critical genes of AD.

**Conclusion:**

Our study identified 16 hub genes correlated to the neuropathological stage and 35 potential biomarkers for the diagnosis of AD. WWTR1 were identified as candidate genes for future studies. This study deepens our understanding of the transcriptomic and functional features and provides new potential diagnostic biomarkers and therapeutic targets for AD.

## Introduction

Alzheimer’s disease (AD) is the most common neurodegenerative disease in the elderly, affecting more than 35.6 million people worldwide (Querfurth and LaFerla, 2010; Kumar et al., 2015). Epidemiological analysis has predicted that the number will rise to 65.7 million in 2030 and approximately 115.4 million in 2050 (Prince et al., 2013). The symptoms usually start with subtle memory loss and gradually progress to affect other cognitive domains as the condition deteriorates, such as language, visuospatial skills, motor skills, executive function, and activities of daily living (McKhann et al., 2011; Huang and Mucke, 2012). As AD usually has concealed onset, most patients with AD are already at an advanced stage at the time of diagnosis. Furthermore, the long-term care and related costs of AD contribute a substantial economic burden to the society and family. It is reported that the global societal cost for dementia is projected to grow to approximately $2 trillion in 2030 (Wimo et al., 2017). Unfortunately, despite recent progress in understanding the neurobiology and pathophysiology of AD so far, no therapeutic strategies can effectively prevent or cure AD. Therefore, research directed toward identifying AD biomarkers is needed for the early diagnosis, prevention, and treatment of AD.

AD is a characteristic “complex” disease resulting from the interaction of genetic and environmental factors. It is known that the primary pathogenesis of AD was β-amyloid (Aβ) abnormal deposition, neurofibrillary tangles induced by phosphorylation of tau proteins, inflammatory response, oxidative stress, and neuronal apoptosis. All these processes involve alterations in the expression and regulation of numerous genes. Studies suggest that genetic factors are estimated to attribute up to 79% to the risk for AD (Wingo et al., 2012). The apolipoprotein E (APOE) ε4 allele has been identified as the most substantial risk factor for AD (Rogaev et al., 1995; Sherrington et al., 1995). Mutations in the genes which enhanced generation and aggregation of Aβ, such as amyloid precursor protein (APP), presenilin (1) (PSEN1), and presenilin (2) (PSEN2), were included in the established genetic causes of familial AD (Sorbi et al., 2001; Tanzi and Bertram, 2005). Moreover, genetic analyses have suggested that the individual differences and complicated pathogenesis of AD may be influenced by multiple genes and their variants involved in numerous biological functions and substantially increase the risk of the disease (Hokama et al., 2014; Stopa et al., 2018). Therefore, identification and comprehensive analyses of potential candidate genes will considerably increase our understanding of the biological mechanisms involved in disease pathogenesis and could potentially be used as diagnostic or predictive biomarkers for AD.

In recent years, bioinformatics analysis is widely applied in molecular biology experiments and clinical practice (Banwait and Bastola, 2015), revealing the key pathways and drug targets in complex diseases (Khan et al., 2018). Thus, joint analysis of the array-based data of AD may be a novel analytical strategy. Our present study aims to reveal the transcriptomic characteristics and identification of novel biomarkers of AD for diagnosis and treatment. We identified co-differentially expressed genes (DEGs) in AD of five microarray datasets in the Gene Expression Omnibus (GEO). Based on the results, we performed a series of analyses, including Gene Ontology (GO), Kyoto Encyclopedia of Genes and Genomes (KEGG) enrichment pathway analysis, protein—protein interaction (PPI) analysis, and least absolute shrinkage and selection operator (LASSO) logistic regression analysis. We identified 18 hub genes and tested their expression levels in different Braak stages. A 35-gene-based diagnosis model was constructed, and then we test the diagnostic values for AD and mild cognitive impairment (MCI). Finally, two key genes were identified by overlapping the 18 hub genes and 35 diagnosis genes. We further explored their correlations with β-secretase activity and Aβ 42 levels. Gene set enrichment analysis (GSEA) was used to explore the potential biological functions of hub genes. Our present study could provide more insights into the molecular mechanism of AD and provided potential biomarker candidates for clinical diagnosis and treatment.

## Materials and Methods

### Data Processing

**FIGURE 1:**
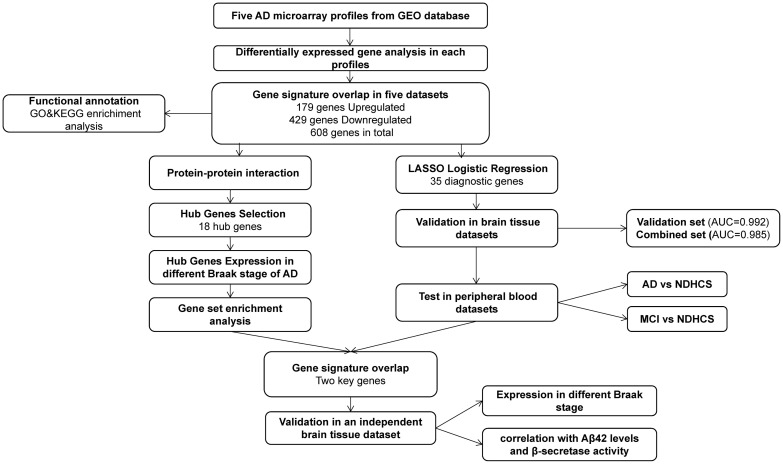
Flowchart for bioinformatics analysis in this study. AD, Alzheimer’s disease; MCI, mild cognitive impairment; NDHCS, non-demented healthy control subjects; AUC, area under the curve; GSEA, gene set enrichment analysis; GEO, Gene Expression Omnibus; GO, Gene Ontology; KEGG, Kyoto Encyclopedia of Genes and Genomes.

GEO^1^ is a public functional genomics data repository of high-throughput gene expression data, chips, and microarrays. According to the following criteria, datasets were considered eligible for our analysis: (1) datasets with AD samples; (2) datasets supported by peer-reviewed PubMed-indexed publications; and (3) studies with information about the technology and platform utilized for studies. We selected 10 datasets (GSE33000, GSE36980, GSE48350, GSE5281, GSE122063, GSE106241, GSE4226, GSE97760, GSE63060, and GSE63061) related to AD for analysis. A total of 757 non-demented healthy control subjects (NDHCS) and 932 AD patients were analyzed. We extracted the whole data of a single study, including all brain regions, for analysis. The data sample collection is shown in Table 1. The flowchart of the study is illustrated in Figure 1.

**TABLE 1:** Dataset characteristics.

Dataset	Platform/technology	No. of samples	Sample source	Age	Gender female:male	Disease stage	Country
GSE33000	GPL4372 (Rosetta/Merck Human 44 k 1.1 microarray)	467 (310 AD, 157 NDHCS)	Prefrontal cortex	AD:(53–100 y); NDHCS:(22–106 y)	209:258	–	United States
GSE36980	GPL6244 [(HuGene-1_0–st) Affymetrix Human Gene 1.0 ST Array (transcript (gene) version)]	79 (32 AD, 47 NDHCS)	Frontal cortex, temporal cortex and hippocampus	AD:(83–105 y); NDHCS:(54–100 y)	42:37	–	Japan
GSE122063	GPL16699 [Agilent–039494 SurePrint G3 Human GE v2 8 × 60 K Microarray 039381 (Feature Number version)]	100 (56 AD, 44 NDHCS)	Frontal cortex, temporal cortex	AD:(63–91 y); NDHCS:(60–91 y)	68:32	–	United States
GSE48350	GPL570 [(HG-U133_Plus_2) Affymetrix Human Genome U133 Plus 2.0 Array]	253 (80 AD, 173 NDHCS)	Hippocampus, entorhinal cortex, superior frontal cortex, post-central gyrus	AD:(60–95 y); NDHCS:(20–99 y)	129:124	Braak stage 0–6	United States
GSE5281	GPL570 [(HG-U133_Plus_2) Affymetrix Human Genome U133 Plus 2.0 Array]	161 (87 AD, 74 NDHCS)	Entorhinal cortex, hippocampus, medial tem poral gyrus, posterior cingulate, superior frontal gyrus and primary visual cortex	AD:(68–97 y); NDHCS:(63–102 y)	58:103	–	United States
GSE106241	GPL24170 [Agilent-044312 Human 8 × 60 K Custom Exon array (Probe Name version)]	60 (60 AD)	Inferior termporal cortex	AD:50–100 y	42:18	Braak stage 0–6	Finland
GSE4226	GPL1211 (NIA MGC, Mammalian Genome Collection)	28 (14 AD, 14 NDHCS)	Peripheral blood mononuclear cells	–	14:14	–	Canada
GSE97760	GPL16699 [Agilent-039494 SurePrint G3 Human GE v2 8 × 60 K Microarray 039381 (Feature Number version)]	19 (9 AD, 10 NDHCS)	Peripheral blood	–	0:19	Advanced AD	United States
GSE63060	GPL6947 (Illumina HumanHT-12 V3.0 expression beadchip)	329 (145 AD, 80 MCI, 104 NDHCS)	Peripheral blood	AD (58–88 y); MCI (63–90 y); NDHCS (52–87 y)	200:129	–	United Kingdom
GSE63061	GPL10558 (Illumina HumanHT-12 V4.0 expression beadchip)	382 (139 AD, 109 MCI, 134 NDHCS)	Peripheral blood	AD (59–95 y); MCI (57–100 y); NDHCS (63–91 y)	231:151	–	United Kingdom

**The first five datasets were used for combined analysis, GSE106241 was used for independent validation analysis, and the last four datasets were used for evaluating the diagnosis model in peripheral blood. AD, Alzheimer’s disease; MCI, mild cognitive impairment; NDHCS, non-demented healthy control subjects. GPL, Gene Expression Omnibus Platform.**

The GSE33000 (platform GPL4372) was composed of postmortem prefrontal cortex (PFC) samples of 157 NDHCS and 310 AD patients with matched genotype and clinical data. The GSE36980 (platform GPL6244) was composed of the frontal cortex (FC), temporal cortex (TC), and hippocampus (HPC) from 47 NDHCS and 32 AD patients. From GSE48350 (platform GPL570), we selected data from the HPC, entorhinal cortex (EC), superior frontal cortex (SFC), and post-central gyrus (PCGY) derived from 253 postmortem brains, among which 80 cases were diagnosed as having AD. The GSE122063 (platform GPL16699) was composed of tissues collected from FC and TC, 44 tissues from NDHCS and 56 from AD patients. The GSE5281 (platform GPL570) was composed of tissues collected by laser capture microscopy (LCM) from 74 NDHCS and 87 AD patients. The brain regions included the EC, HPC, medial temporal gyrus, posterior cingulate, SFC, and primary visual cortex. The GSE106241 was enrolled for independent external validation. In this dataset, 60 human temporal cortical tissue samples were included and divided into seven groups based on Braak staging.

Gene expression profiles of peripheral blood were obtained from GSE97760, GSE4226, GSE63060, and GSE63061. Study subjects from GSE97760 (platform GPL16699) were all female, including nine subjects with advanced AD and 10 age-matched NDHCS. The GSE4226 (platform GPL1211) was composed of peripheral blood mononuclear cells from 14 NDHCS and 14 AD patients. Datasets GSE63060 and GSE63061 were composed of MCI patients, AD patients, and NDHCS. There are 329 samples (145 AD, 80 MCI, and 104 NDHCS) in GSE63060 and 382 samples (139 AD, 109 MCI, 134 NDHCS) in GSE63061. Three borderline MCI samples, one NDHCS-to-AD sample, one MCI-to-NDHCS sample, and one other sample, were excluded from GSE63061.

### Identification of Consensus DEGs

As single datasets and few samples may weaken the credibility of the results, data integration is necessary to look for findings supported by several pieces of evidence and investigate the complex genetic mechanisms (Pineda et al., 2015). Therefore, five brain tissue datasets (GSE33000, GSE36980, GSE48350, GSE5281, and GSE122063) were selected to identify consensus DEGs. We used the impute package to supplement missing data (Troyanskaya et al., 2001). Then, the normalizeBetweenArrays function in the limma package was used to normalize gene expression. Next, we performed the differential analysis in each of the datasets. We screened DEGs by comparing AD tissues to NDHCS tissues in the R computing environment using the limma package (Ritchie et al., 2015). DEGs were determined by | Log 2 FC| > 0, adjusted *p*-value < 0.05. Volcano plots were generated using ggplot 2 in R. In order to obtain a consensus of DEGs, Venn analysis was performed using Draw Venn Diagram, a Web-based tool,^2^ to identify common DEGs from the five datasets. The heatmap of the consensus DEGs was drawn using the R pheatmap package. We performed the batch correction, followed by normalization between arrays to remove the heterogeneity among multiple microarray datasets using sva and limma packages (Leek et al., 2012). Finally, principal component analysis (PCA) was performed to compare the difference of consensus DEGs between AD and NDHCS groups in different brain regions.

### GO Enrichment and KEGG Pathway Analysis of the DEGs

GO enrichment analyses were performed in R using the function of clusterProfiler14. Metascape^3^ was used to perform the KEGG pathway analysis. Functional and pathway enrichment analyses were conducted separately for upregulated and downregulated genes. In this analysis, a *p*-value < 0.05 was considered significant for the screening of significant GO terms and KEGG pathways. Furthermore, we performed the differential analysis separately in 10 brain regions. DEGs were determined by | Log 2 FC| > 0, adjusted *p*-value < 0.05. The top 100 upregulated DEGs and top 100 downregulated DEGs of each brain region were used for GO enrichment analyses. Finally, we take the intersection of pathways of each brain region to identify common and specific dysregulated pathways.

### PPI Network Construction, Hub Gene Selection, and Hub Gene Expression in Different Braak Stages of AD

To further explore the interactions among DEGs, PPI network analysis was performed using the online database STRING with an interaction score of 0.4 as the threshold. Next, we utilized Cytoscape (version 3.7.1) to construct and visualize the main regulatory network. We used cytoHubba, a plugin of Cytoscape, to select the hub genes in the PPI network. Five methods (degree, maximum neighborhood component (MNC), radiality centrality, stress centrality, closeness centrality) were used to sequence and evaluate central genes (Chin et al., 2014). The five ranked methods selected the top 20 hub genes. Venn analysis was performed to identify central genes by overlapping the top 20 genes. Finally, we validate hub gene expressions in different Braak stages using the HPC and superior frontal cortex (SPF) samples in the dataset GSE48350. Overall differences between groups were tested with the Kruskal—Wallis (K-W) test, and differences between groups were compared by the Wilcox test. The boxplot was drawn by R package ggplot 2. The top 10 hub genes were also further evaluated based on the difference in the gene expression of GSE48350.

### Identification of Potential Biomarkers of AD Using LASSO Logistic Regression

The LASSO, a penalized shrunken regression method, has a strong predictive value and low correlation and is applied to select the best features for high-dimensional data. The samples from five brain tissue datasets were randomly assigned to the training set (30%) and validation set (70%). The expression profiles of consensus DEGs were extracted and fit into LASSO logistic regression by the glmnet package. In order to evaluate the ability of the LASSO model to identify AD, receiver operating characteristic (ROC) analysis was completed using the package of pROC in the validation set and combined set (Robin et al., 2011). The area under the curve (AUC) was calculated, and AUC values close to 1 (AUC > 0.7) refer to good classifier models. We also investigated the diagnosis effect of the top 10 hub genes in the combined set.

### Evaluate the Diagnosis Model in Peripheral Blood Datasets

As it is hard to obtain brain tissues for diagnosis in clinical practice, we attempted to enroll independent peripheral blood datasets to evaluate the clinical utility of our diagnosis model. We performed ROC analyses in GSE4226 and GSE97760 to examine the ability to differentiate AD from NDHCS. The datasets GSE63060 and GSE63061 were used to verify the accuracy of the model to differentiate MCI and AD from NDHCS. ROC curves were plotted using the “pROC” package.

### GSEA and Independent Validation Analysis

GSEA was performed to identify biological process (BP) GO terms of the top 10 hub genes that may be correlated to AD in GSE48350 datasets. We performed GSEA using the R package clusterProfiler for analysis. The c5.bp.v 7.0.symbols.gmt datasets in the MsigDB V 6.2 database^4^ were used as reference gene sets, and those with an adjusted *p*-value < 0.05 after 1,000 permutations were considered significantly enriched gene sets (Subramanian et al., 2005). We determined the key genes by overlapping the hub genes selected from the PPI network and potential diagnosis genes identified from the LASSO regression model. Next, we enrolled another independent dataset (GSE106241) and compared the expression level of key genes in different Braak stages using the K-W test. Moreover, we investigated their associations with β-secretase activity and Aβ 42 levels in AD samples from GSE106241 using the Spearman correlation analysis. *p*-values less than 0.05 (*p* < 0.05) were considered significant. The violin plots and correlation analysis in this section were all generated in R 3.6.3.

## Results

### Identification of Consensus DEGs

**FIGURE 2:**
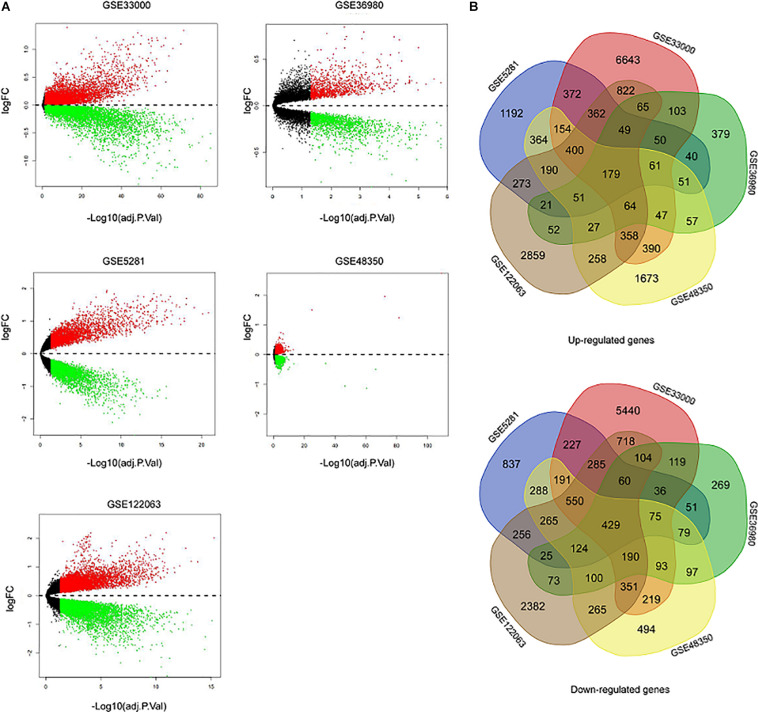
Identification of DEGs between AD and NDHCS samples. **(A)** The volcano plot of the genes in the five datasets. **(B)** Venn diagram analysis of common downregulated DEGs and common upregulated DEGs. AD, Alzheimer’s disease; NDHCS, non-demented healthy control subjects.

Five microarray datasets, including GSE33000, GSE36980, GSE48350, GSE5281, and GSE122063, were downloaded from the National Center of Biotechnology Information-GEO (NCBI-GEO). Details of the five datasets are presented in Table 1. A total of 1,060 samples (495 NDHCS subjects and 565 AD patients) were available for DEG analysis, including microarray data from 10 brain regions: the EC, FC, HPC, medial temporal gyrus, PCGY, posterior cingulate, PFC, primary visual cortex, SFC, and TC. After background correction and normalization, we used the limma package to identify DEGs between NDHCS and AD samples of GEO data. Gene difference analysis found that there were 19,206 DEGs in GSE33000, 3,220 DEGs in GSE36980, 8,134 DEGs in GSE48350, 7,587 DEGs in GSE5281, and 12,207 DEGs in GSE122063 compared with AD patients and NDHCS (Supplementary Table 1). Volcano plots in Figure 2A show the number of DEGs identified from each of the five datasets. Subsequently, we intersected these DEGs from the five datasets and finally identified 608 common DEGs, of which 179 DEGs were upregulated and 429 DEGs were downregulated (Figure 2B and Supplementary Table 1). To compare the DEGs between the AD and NDHCS groups, the heatmap showed the expression of common DEGs from five datasets (Supplementary Figure 1). PCA revealed that the expression of common DEGs differed significantly between NDHCS and AD samples in each brain region, indicating that the DEGs we found were common core genes in AD (Supplementary Figure 2).

### GO Enrichment and KEGG Pathway Analysis of the DEGs

**FIGURE 3:**
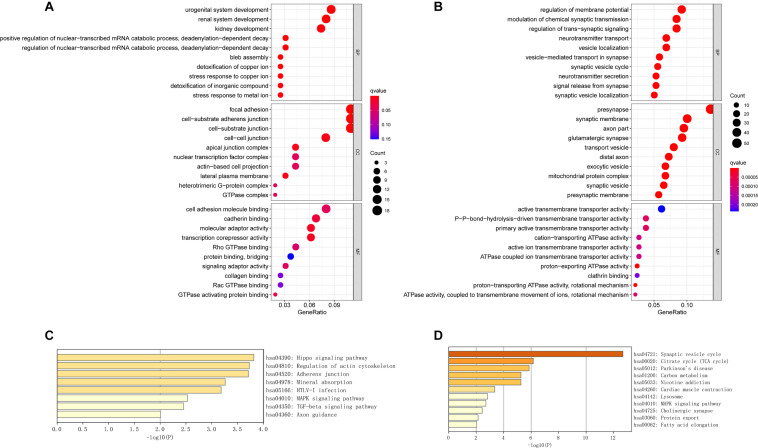
The GO analysis and KEGG pathway analysis of upregulated and downregulated DEGs. **(A)** The GO analysis of common upregulated DEGs. **(B)** The GO analysis of common downregulated DEGs. **(C)** The KEGG pathway analysis of common upregulated DEGs. **(D)** The KEGG pathway analysis of common downregulated DEGs.

We performed GO term, KEGG pathway, and functional enrichment analyses to explore the potential biological functions of the common DEGs. The GO annotation results include BP, molecular function (MF), and cellular component (CC). The results revealed that the BP primarily associated with the upregulated genes, including the regulation of the nuclear-transcribed mRNA catabolic process, bleb assembly, detoxification of copper ion, stress response to copper ion, detoxification of inorganic compound, and stress response to metal ion. For CC enrichment analysis, the results showed that upregulated genes significantly took part in the focal adhesion, cell—substrate adherens junction, cell—substrate junction, and cell—cell junction. For MF analysis, upregulated genes are mainly enriched in cell adhesion molecule binding, cadherin binding, molecular adaptor activity, and transcription corepressor activity (Figure 3A). For downregulated genes, regulation of membrane potential, modulation of chemical synaptic transmission, regulation of transsynaptic signaling, and neurotransmitter transport were dominant BPs. For CC enrichment analysis, downregulated genes mainly take part in presynapse, synaptic membrane, axon part, and glutamatergic synapse. In the enrichment analysis of MF, the downregulated genes mainly revolved in active transmembrane transporter activity, P—P-bond-hydrolysis-driven transmembrane transporter activity, and primary active transmembrane transporter activity (Figure 3B). The KEGG pathway analysis showed that the upregulated genes were significantly enriched in the Hippo signaling pathway, regulation of actin cytoskeleton, adherens junction, mineral absorption, MAPK signaling pathway, and TGF-beta signaling pathway, while the downregulated DEGs were mainly enriched in the synaptic vesicle cycle, citrate cycle (TCA cycle), Parkinson’s disease, lysosome, MAPK signaling pathway, and cholinergic synapse. The KEGG pathway enrichment analysis results are illustrated in Figures 3C,D. The complete results of GO and KEGG analyses can be found in Supplementary Table 2.

### Specific Dysregulated Pathways for Each Brain Region

We performed GO enrichment analyses using the top 100 upregulated DEGs and top 100 downregulated DEGs of each brain region. Then we identified the specific dysregulated pathways of each brain region. There were two specific pathways in the EC and the PCGY, 20 pathways in the FC, 12 pathways in the HPC, 145 pathways in the medial temporal gyrus, 38 pathways in the posterior cingulate, 47 pathways in the primary visual cortex, four pathways in the TC, and 58 pathways in the PFC (Supplementary Table 2). There was no specific pathway in the SFC. The HPC was associated with the neuron projection organization. The medial temporal gyrus was associated with neuron projection maintenance, neurotransmitter receptor transport to the plasma membrane, neurotransmitter receptor transport to the postsynaptic membrane, and response to Aβ. The dysregulated genes in the posterior cingulate were significantly enriched in pathways, including the branching morphogenesis of a nerve, glutamate metabolic process, positive regulation of synaptic transmission, and glutamatergic pathway. In the primary visual cortex, the dysregulated genes were involved in neuromuscular synaptic transmission, neuroinflammatory response, and cell aging. The neurotransmitter reuptake was associated with TC. Peripheral nervous system development and nerve development were significantly enriched pathways in PFC.

### PPI Network Construction, Hub Gene Selection, and Hub Gene Expression in Different Braak Stages of AD

**FIGURE 4:**
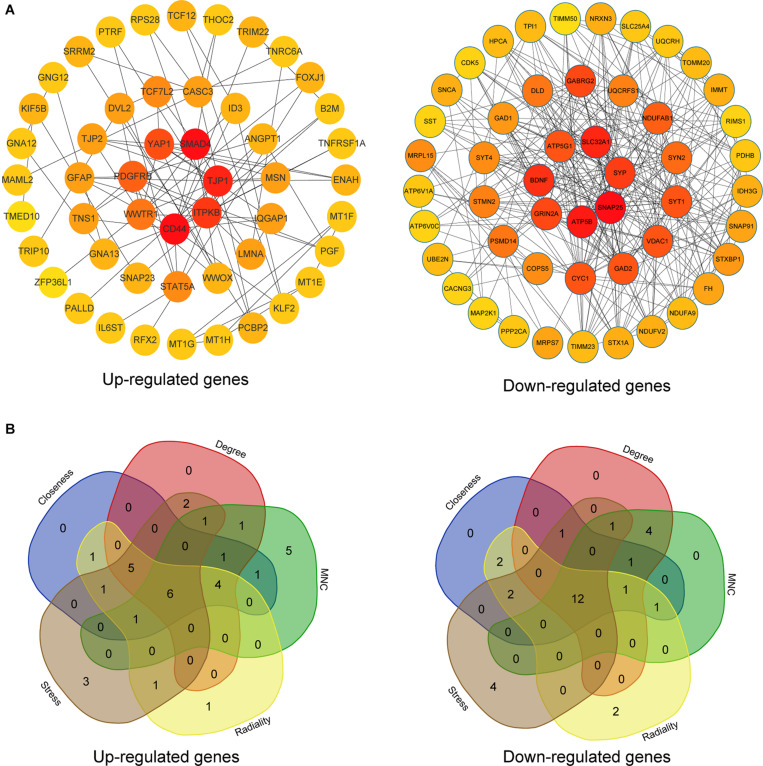
PPI network and hub gene selection. **(A)** The top 50 hub genes in the PPI network of the upregulated and downregulated DEGs according to node degree. **(B)** Hub genes were identified by overlapping the first 20 genes in the five classification methods of cytoHubba.

**FIGURE 5:**
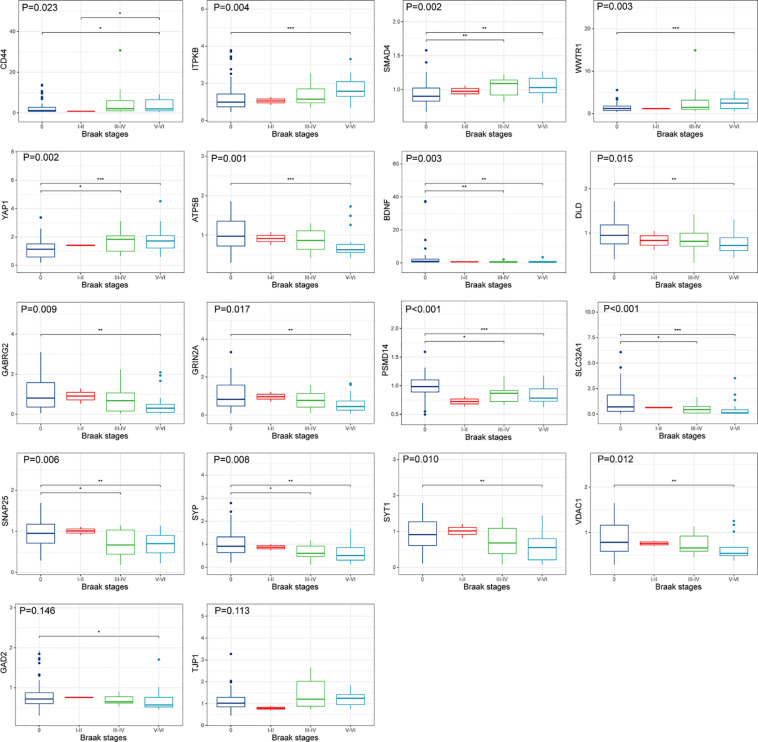
Hub gene expression in different Braak stages of AD. The upper, middle, and lower horizontal lines of the box represent the upper, median, and lower quartiles, respectively. Overall differences between groups were tested with the K-W test. Asterisks indicate significant vs. Braak 0 groups; *p < 0.05; **p < 0.01; ***p < 0.001 (dataset GSE48350; *n* = 91 for NDHCS; *n* = 2 for Braak stages I and II; *n* = 15 for Braak stages III and IV; *n* = 21 for Braak stages V and VI). AD, Alzheimer’s disease; NDHCS, non-demented healthy control subjects; HPC, hippocampus; PFC, prefrontal cortex.

The upregulated and downregulated DEGs were uploaded into the online tool STRING^5^ to gain PPI information separately. Based on the degree of connectivity, we constructed the PPI network and selected the top 50 hub genes, and the result was visualized by Cytoscape (Figure 4A). Next, we used cytoHubba to choose hub genes. According to the five classification methods in cytoHubba, we selected the top 20 hub genes, as shown in Supplementary Table 3. Finally, six upregulated and 12 downregulated central genes were identified by overlapping the first 20 genes (Figure 4B and Supplementary Table 3). Then, we used samples in the dataset GSE48350 to explore whether the expression levels of these central genes varied in different Braak stages in the HPC and SPF (Supplementary Table 4). We found that five hub genes were upregulated and 11 hub genes were downregulated as AD progressed (*p* < 0.05). Predominantly, SMAD 4 and YAP 1 were significantly upregulated in Braak III and IV and Braak V and VI compared with Braak 0. Brain-derived neurotrophic factor (BDNF), PSMD14, SLC32A1, SNAP25, and SYP were identified to be downregulated in Braak III and IV and Braak V and VI compared with Braak 0 (Figure 5).

### Identification of Potential Biomarkers of AD Using LASSO Logistic Regression

**FIGURE 6:**
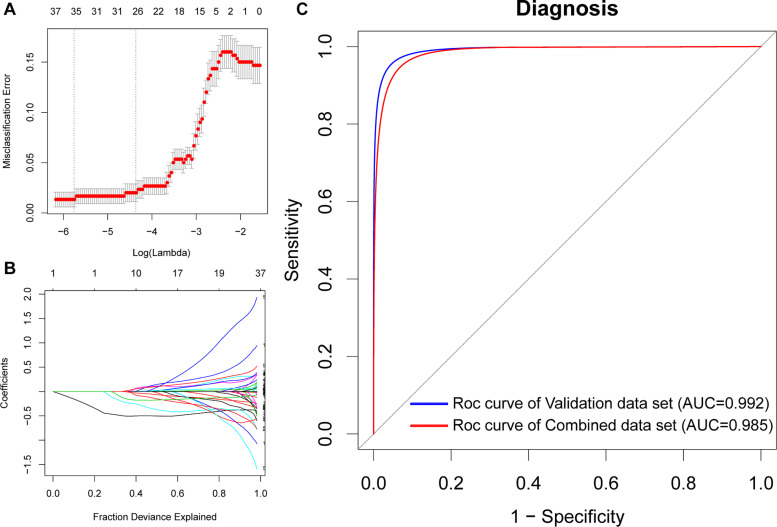
Gene selection through the LASSO model. **(A)** Tenfold cross-validation for tuning parameter (lambda) selection in the LASSO regression model. The vertical lines were drawn at the optimal values by the minimum criteria and the 1 – SE criteria. **(B)** The LASSO coefficient profiles of the 608 DEGs. **(C)** ROC curve analysis of the validation dataset and combined dataset.

To identify potential biomarkers for AD, we extracted the expression profile of the DEGs and fit them into LASSO logistic regression. We separated all samples (565 AD samples and 495 NDHCS) into training and validation cohorts (Supplementary Table 5). Thirty-five potential predictors in the training cohort were identified and were features with nonzero coefficients in the LASSO logistic regression model (Figures 6A,B and Table 2). Next, we evaluated the ability of the LASSO regression model in differentiating between AD and NDHCS, suggesting that the AUC of the 35-gene-based model was 0.992 in the validation set and 0.985 in the combined set (Figure 6C). The results indicate that our 35-gene-based diagnosis model can correctly classify AD samples and NDHCS in brain tissues. The diagnosis effect of the top 10 hub genes was also investigated and presented in Supplementary Figure 3. Only BDNF had the ability to differentiate AD from NDHCS (AUC = 0.703).

**TABLE 2:** The list of 35 potential biomarkers of AD using LASSO logistic regression.

UniProt ID	Protein name	Gene name
P21291	Cysteine- and glycine-rich protein 1	CSRP1
Q01628	Interferon-induced transmembrane protein 3	IFITM3
P13640	Metallothionein–1 G	MT1G
P04732	Metallothionein–I E	MT1E
Q9Y2D9	Zinc finger protein 652	ZNF652
Q7Z3K3	Pogo transposable element with ZNF domain	POGZ
Q9GZV5	WW domain-containing transcription regulator protein 1	WWTR1
Q9NZH0	G-protein-coupled receptor family C group 5 member B	GPRC5B
P23949	mRNA decay activator protein ZFP36L2	ZFP36L2
Q9BX66	Sorbin and SH3 domain-containing protein 1	SORBS1
Q8N8S7	Protein enabled homolog	ENAH
Q68DX3	FERM and PDZ domain-containing protein 2	FRMPD2
P61278	Somatostatin	SST
P23560	Brain-derived neurotrophic factor	BDNF
Q8N4V2	Synaptic vesicle 2-related protein	SVOP
Q8N967	Leucine-rich repeat and transmembrane domain-containing protein 2	LRTM2
Q99259	Glutamate decarboxylase 1	GAD1
Q16566	Calcium/calmodulin-dependent protein kinase type IV	CAMK4
O95206	Protocadherin-8	PCDH8
P61088	Ubiquitin-conjugating enzyme E2 N	UBE2N
O43768	Alpha-endosulfine	ENSA
Q9UIL1	Short coiled-coil protein	SCOC
Q13530	Serine incorporator 3	SERINC3
Q9Y5V3	Melanoma-associated antigen D 1	MAGED1
Q96F83	Clathrin-binding box of aftiphilin-containing protein 1	C14orf79
P80723	Brain acid soluble protein 1	BASP1
Q15904	V-type proton ATPase subunit S1, V-ATPase subunit S 1	ATP6AP1
O95197	Reticulon-3	RTN3
Q96CW1	AP-2 complex subunit mu	AP2M1
P17643	5,6-Dihydroxyindole-2-carboxylic acid oxidase	TYRP1
P50613	Cyclin-dependent kinase 7	CDK7
Q8N100	Protein atonal homolog 7	ATOH7
Q9Y6G3	39S ribosomal protein L 42, mitochondrial, L 42 mt, MRP-L 42	MRPL42
O00744	Protein Wnt-10 b	WNT10B
P84074	Neuron-specific calcium-binding protein hippocalcin	HPCA

### Evaluation of the Diagnosis Model in Peripheral Blood Datasets

**FIGURE 7:**
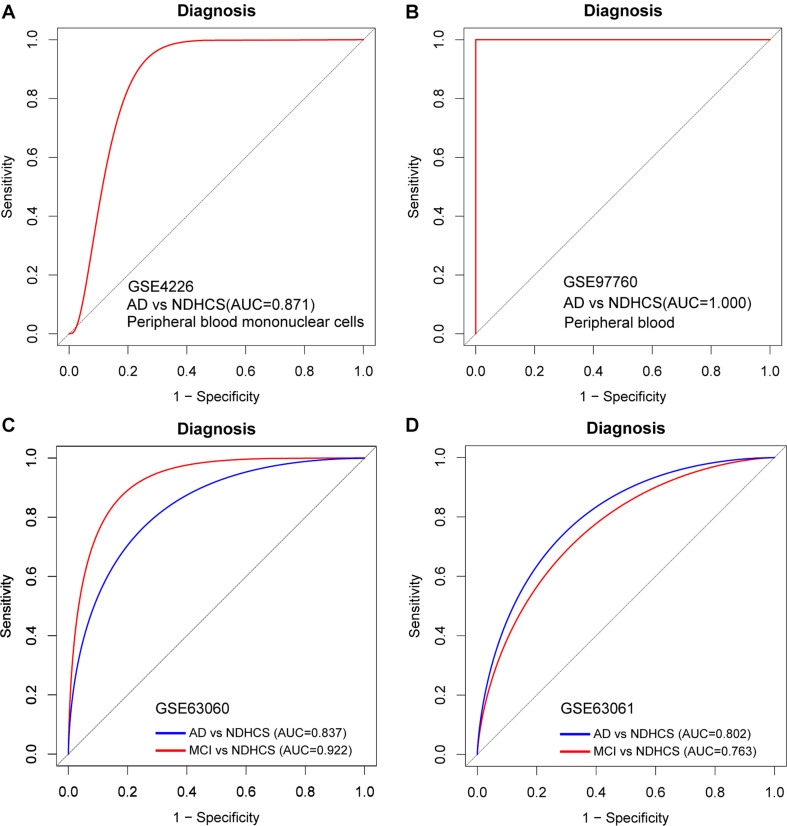
ROC curve analysis in blood datasets. **(A)** ROC curves of peripheral blood mononuclear cell data from GSE4226. **(B)** ROC curves of peripheral blood data from GSE97760. **(C)** ROC curve analysis of GSE63060. **(D)** ROC curve analysis of GSE63061. AD, Alzheimer’s disease; MCI, mild cognitive impairment; NDHCS, non-demented healthy control subjects; AUC, area under the curve.

To further discover whether this model is worth using in clinical practice, we test our diagnosis model on four independent peripheral blood datasets (Supplementary Table 5). In the GSE4226, composed of peripheral blood mononuclear cells, only 16 genes of the 35-gene-based model were covered. The ROC analysis was also conducted based on the 16 genes, and the AUC was 0.871 (Figure 7A). We applied the 35-gene-based model to GSE97760, which included patients with advanced AD and NDHCS and showed the perfect discrimination ability (Figure 7B, AUC = 1.000). We also found that our diagnosis model had the ability to differentiate MCI and AD from NDHCS in the blood datasets. In GSE63060 (30 genes of the 35-gene-based model were covered), the AUC for differentiating MCI and NDHCS is 0.922, and the AUC for AD and NDHCS is 0.837 (Figure 7C). In GSE63061 (28 genes of the 35-gene-based model were covered), AUC for MCI and NDHCS is 0.763, and AUC for AD and NDHCS is 0.802 (Figure 7D). Conclusively, the results indicate that our 35-gene-based diagnosis model can classify AD and MCI from NDHCS in peripheral blood.

### GSEA and Independent Validation Analysis

**FIGURE 8:**
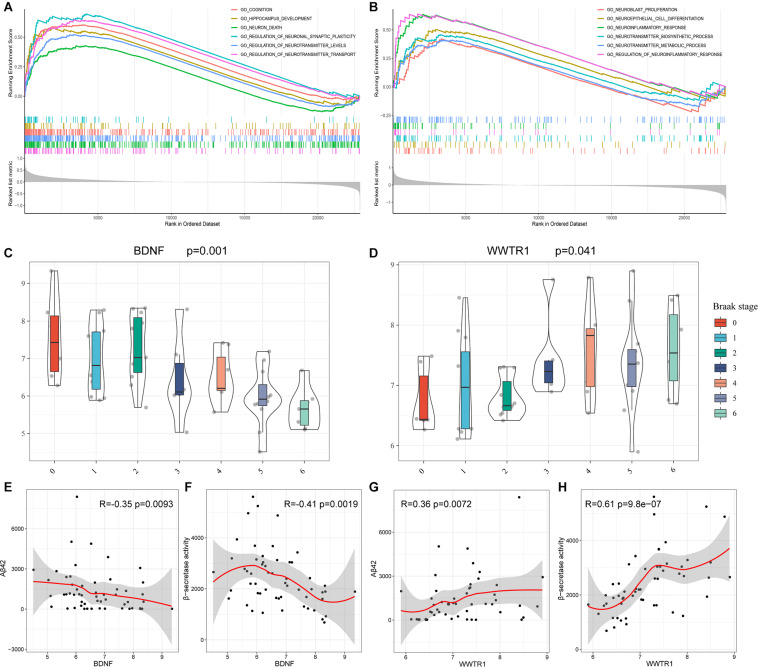
GSEA based on GSE48350 and independent validation analysis based on GSE106241. **(A)** BDNF. **(B)** WWTR1. **(C)** BDNF expression levels in the Braak stages 0–6. **(D)** WWTR1 expression levels in Braak stages 0–6. **(E)** Correlation between BDNF and Aβ 42 levels. **(F)** Correlation between BDNF and β-secretase activity. **(G)** Correlation between WWTR1 and Aβ 42 levels. **(H)** Correlation between WWTR1 and β-secretase activity. *p*-values < 0.05 were considered to be statistically significant.

To gain new insights into the biological functions of the top 10 genes, we performed GSEA to identify the potential BPs between AD and NDHCS subjects. As indicated in Figures 8A,B, the BDNF was associated with cognition, HPC development, neuron death, regulation of neuronal synaptic plasticity, regulation of neurotransmitter levels, and transport. BPs such as neuroblast proliferation, neuroepithelial cell differentiation, neurotransmitter biosynthetic process and metabolic process, neuroinflammatory response, and regulation of neuroinflammatory response were associated with WW domain-containing transcription regulator protein 1 (WWTR1). The rest of the top 10 hub genes were also involved in several neuron-related pathways, including axon development (SMAD4, SLC32A1, and YAP1), neuron death (SNAP25 and ATP5B), and synapse organization (SMAD4 and SLC32A1). The results are shown in Supplementary Figure 4.

BDNF and WWTR1 were identified as key genes by overlapping the 18 hub genes in the PPI network and 35 potential predictors selected from the LASSO regression model. To confirm the result, we enrolled another independent dataset (GSE106241) to conduct validation analysis (Supplementary Table 5). As shown in Figures 8C,D, BDNF and WWTR1 showed significant differences among the different Braak stages (*p* = 0.001 and 0.041, respectively). Moreover, we found that BDNF was negatively associated with Aβ 42 levels and β-secretase activity (*R* = –0.35 and *R* = –0.42, respectively, Figures 8E,F). WWTR1 was positively associated with Aβ 42 levels and β-secretase activity (*R* = 0.36 and *R* = 0.61, respectively, Figures 8G,H). This result further proves that the two genes are essential and involved in the pathology of AD.

## Discussion

Bioinformatics analysis has developed rapidly and applied to many diseases in recent decades, revealing the complex pathogenesis and identifying new biomarkers for diagnosis and treatment (Ali et al., 2018). Nevertheless, integrated bioinformatics analysis has not yet been systematically used in AD. Previous researches were usually based on single datasets and few samples that may weaken the credibility of the results. However, our current study has recruited five open public datasets for DEGs to significantly improve the number of samples (495 NDHCS vs. 656 AD samples). Thus, our study provides more credible and trustworthy results. We performed a series of integrative analyses based on DEGs, including GO and KEGG enrichment analyses, a constructed PPI network, LASSO logistic regression, and GSEA. In this way, we provide valuable clues for investigating the molecular mechanisms underlying the initiation and development of AD.

The KEGG pathway enrichment analysis showed significant enrichment in pathways including the Hippo signaling pathway, the TGF-beta signaling pathway, the MAPK signaling pathway, the synaptic vesicle cycle, lysosome, and the cholinergic synapse. A recent study confirmed that the Hippo pathway is associated with the pathogenesis of AD. The precursor of Aβ can promote the nuclear translocation of FOXO 3a by inducing MST1-dependent phosphorylation of Foxo 3a. The MST-Foxo pathway, which is considered a branch of the Hippo pathway, activates a proapoptotic member of the Bcl-2 family and triggers an intrinsic apoptotic pathway, resulting in neuronal death (Wang and Wang, 2016). Regarding the TGF-beta signaling pathway, previous studies showed that the expression of TGF-β 1 and TGF-β 2 increased in the brains of patients with AD (Zetterberg et al., 2004). Given the current evidence of microglial dysfunction in neurodegeneration, we speculate that changes in brain TGF-β signaling in AD could alter microglial state and trigger their pathogenic functions (Salter and Stevens, 2017). It is well documented that lysosomal dysfunction is a prominent feature in AD brains, resulting in a failure to clear accumulated protein aggregates and contributes to the process of the pathogenesis of AD (Fraldi et al., 2016). Similarly, the MAPK signal pathways are activated in vulnerable brain regions of AD patients and are involved in the progress of AD (Zhu et al., 2002; Guillot et al., 2016). Therefore, the MAPKs have been proposed as therapeutic targets for AD. Previous studies also confirm that cholinergic transmission impairments are correlated with the neuropathological stage of AD (Amberla et al., 1993). A decrease in the cholinergic activity and disruption of synaptic function contribute to memory impairment (Sayer et al., 2004). Furthermore, the GO analysis indicated that the DEGs are involved in a wide range of BPs and have different MFs. We also found several specific dysregulated pathways in each brain region. Our results indicated that the changes in BPs, CCs, MFs, and pathways might play critically important roles in the pathogenesis of AD.

We identified 18 hub genes by overlapping five sequencing methods in cytoHubba, of which 16 hub genes were significantly dysregulated as AD progressed. Some of these genes have been previously reported to be associated with AD. For example, the reduction of SNAP25 causes postsynaptic loss and learning and memory impairment (Ren et al., 2018). A recent study has demonstrated that SNAP25 is an effective biomarker for predicting AD 5–7 years before cognitive impairment (Jia et al., 2020). SYP can affect the synaptic structure and neurotransmitter release to regulate synaptic plasticity (Zhu et al., 2019). The dysfunction of ATP5B is associated with neurofibrillary tangle burden in the AD brain and with cognition (Wang et al., 2017). The decrease of BDNF correlates with the neuropathological stage of AD (Laske et al., 2006). Further studies are required to investigate their features, functions, and mechanisms.

In the present study, we constructed a 35 gene-based LASSO model, which can accurately predict AD in both validation and combined brain tissue datasets. Among the 35 genes, previous studies have reported that the expressions of IFITM3 (Correani et al., 2017), SORBS1 (Blalock et al., 2004), ENAH (de Oliveira-Júnior et al., 2015), SST (Solarski et al., 2018), ENSA (Boettcher et al., 2008), C14orf40 (Chung et al., 2018), BASP1 (Zhou et al., 2020), RTN3 (Zou et al., 2018), CDK7 (Zhu et al., 2000), and HPCA (Jiang et al., 2016) were associated with AD or have functions in neural tissue, indicating possible therapeutic targets. For example, IFITM3 is a reliable biomarker of the inflammatory microglial phenotype in AD damaged tissues (Correani et al., 2017), and the expression of SORBS1 is higher, while the expression of ENSA is lower in the brain of patients with AD (Blalock et al., 2004; Boettcher et al., 2008). SST interferes with Aβ fibrillization and promotes the formation of Aβ assemblies (Solarski et al., 2018). However, the molecular mechanism of these 35 genes contributing to AD pathogenesis is still poorly understood, and further exploration of potential mechanisms may be valuable.

To assess and confirm its clinical application value, we validated our gene signature on peripheral blood. According to ROC curves, the 35-gene-based model is able to distinguish MCI and AD samples from NDHCS samples in the blood. Especially in GSE97760, the model shows the perfect ability to select advanced AD patients (AUC = 1.000). Our results indicate that this diagnosis model could be beneficial for clinical applications. It is known that the blood—brain barrier (BBB) controls substance exchange strictly between the brain and blood. However, studies indicate that the breakdown of BBB could enhance the movement of proteins between the brain and blood in either direction (Zipser et al., 2007). Thus, some proteins in the blood might be associated with AD pathology. Recent studies have also adopted the strategy to integrate brain and blood datasets to identify potential AD biomarkers (Yao et al., 2018; Zhu et al., 2020). Our results shed new light on diagnosis biomarker identification. Further large-sample studies with a different analysis in the blood are required to confirm our results.

This study detected two key genes, BDNF and WWTR1, as potential biomarkers for clinical diagnosis and therapeutic monitoring in AD. Both of them have been identified as hub genes in the PPI network and varied significantly in the different neuropathological stages of AD. They were also selected as potential diagnostic biomarkers from LASSO logistic regression. GSEA suggests that the BDNF and WWTR1 could function as key players in a broad array of essential signaling pathways. Moreover, an independent validation showed that BDNF and WWTR1 are associated with the Braak stage, Aβ 42 levels, and β-secretase activity.

BDNF, located on chromosome 11 p 14, is reported to play an essential role in regulating neurodevelopment, promoting neuronal survival, and supporting basal forebrain cholinergic projections to the HPC and neocortex (Marmigère et al., 2003; Huang et al., 2007). Previous studies indicate that BDNF depletion led to an increase in cortical amyloid plaque numbers and size (Braun et al., 2017). It has also been reported that the expression of BDNF decreased in the brain tissue of patients with AD (Connor and Dragunow, 1998; Fields et al., 2014). This is consistent with our results that BDNF is negatively correlated with the Braak stage, and GSEA suggested that it is involved in HPC development, cognition, neuron death, and neurotransmitter regulation. Thus, the downregulation of BDNF may play a crucial role in the pathogenesis of AD. WWTR1 is another potential diagnosis biomarker and may contribute to the development of AD. Functional enrichment analysis showed that WWTR1 was significantly involved in the neuroinflammatory response and neurotransmitter biosynthetic and metabolic pathways. Previous research indicates that WWTR1 is playing a crucial role in the Hippo and TGF-beta pathways, which is associated with the progress of AD (Lei et al., 2008; Deiana et al., 2018). However, the mechanisms of WWTR1 and AD remain undefined. More research is needed to elucidate the functions and underlying mechanisms of WWTR1 and AD. Although we enrolled an additional dataset for external validation, high-quality validation experiments are still required to prove the value of BDNF and WWTR1 in AD pathology.

## Conclusion

In conclusion, we identified 608 consensus DEGs, several dysregulated pathways, and 16 hub genes associated with AD progress by a series of bioinformatics analyses. The diagnostic model of 35 genes was constructed, which has a high AUC value in not only brain tissue but also peripheral blood. BDNF and WWTR1 were identified as candidate genes for future molecular studies. Our current study deepens our understanding of underlying molecular mechanisms in AD and provides new potential diagnostic and therapeutic biomarkers.

## Data Availability Statement

The datasets generated for this study can be found in the online repositories. The names of the repository/repositories and accession number(s) can be found in the article/Supplementary Material.

## Author Contributions

WuY and YL designed the study. WuY, WeY, and YY performed the statistical analysis and wrote the manuscript. All authors reviewed the manuscript and gave the final approval for publication.

## Conflict of Interest

The authors declare that the research was conducted in the absence of any commercial or financial relationships that could be construed as a potential conflict of interest.

## Footnotes

1 http://www.ncbi.nlm.nih.gov/geo

2 http://bioinformatics.psb.ugent.be/webtools/Venn/

3 http://metascape.org

4 http://software.broadinstitute.org/gsea/msigdb/

5 http://string-db.org

## References

[B1] Ali A. Junaid M. Khan A. Kasushik A. C. Wei D. Q. (2018). Identification of novel therapeutic targets in myelodysplastic syndrome using protein-protein interaction approach and neural networks. *J. Comput. Sci. Syst. Biol.* 11:2. 10.4172/jcsb.1000270

[B2] Amberla K. Nordberg A. Viitanen M. Winblad B. (1993). Long-term treatment with tacrine (THA) in Alzheimer’s disease–evaluation of neuropsychological data. *Acta Neurol. Scand. Suppl.* 149 55–57. 10.1111/j.1600-0404.1993.tb04257.x 8128841

[B3] Banwait J. K. Bastola D. R. (2015). Contribution of bioinformatics prediction in microRNA-based cancer therapeutics. *Adv. Drug Deliv. Rev.* 81 94–103. 10.1016/j.addr.2014.10.030 25450261 PMC4277182

[B4] Blalock E. M. Geddes J. W. Chen K. C. Porter N. M. Markesbery W. R. Landfield P. W. (2004). Incipient Alzheimer’s disease: microarray correlation analyses reveal major transcriptional and tumor suppressor responses. *Proc. Natl. Acad. Sci. U.S.A.* 101 2173–2178. 10.1073/pnas.0308512100 14769913 PMC357071

[B5] Boettcher J. M. Hartman K. L. Ladror D. T. Qi Z. Woods W. S. George J. M. (2008). Membrane-induced folding of the cAMP-regulated phosphoprotein endosulfine-alpha. *Biochemistry* 47 12357–12364. 10.1021/bi801450t 18973346

[B6] Braun D. J. Kalinin S. Feinstein D. L. (2017). Conditional depletion of hippocampal brain-derived neurotrophic factor exacerbates neuropathology in a mouse model of Alzheimer’s disease. *ASN Neuro* 9:1759091417696161. 10.1177/1759091417696161 PMC5415058 28266222

[B7] Chin C. H. Chen S. H. Wu H. H. Ho C. W. Ko M. T. Lin C. Y. (2014). cytoHubba: identifying hub objects and sub-networks from complex interactome. *BMC Syst. Biol.* 8(Suppl. 4):S11. 10.1186/1752-0509-8-S4-S11 25521941 PMC4290687

[B8] Chung J. Wang X. Maruyama T. Ma Y. Zhang X. Mez J. (2018). Genome-wide association study of Alzheimer’s disease endophenotypes at prediagnosis stages. *Alzheimers Dement.* 14 623–633. 10.1016/j.jalz.2017.11.006 29274321 PMC5938137

[B9] Connor B. Dragunow M. (1998). The role of neuronal growth factors in neurodegenerative disorders of the human brain. *Brain Res. Brain Res. Rev.* 27 1–39. 10.1016/s0165-0173(98)00004-6 9639663

[B10] Correani V. Di Francesco L. Mignogna G. Fabrizi C. Leone S. Giorgi A. (2017). Plasma membrane protein profiling in beta-amyloid-treated microglia cell line. *Proteomics* 17:1600439. 10.1002/pmic.201600439 28815942

[B11] de Oliveira-Júnior L. C. Araújo Santos Fde A. Goulart L. R. Ueira-Vieira C. (2015). Epitope fingerprinting for recognition of the polyclonal serum autoantibodies of Alzheimer’s disease. *Biomed. Res. Int.* 2015:267989. 10.1155/2015/267989 PMC4568325 26417591

[B12] Deiana M. Dalle Carbonare L. Serena M. Cheri S. Parolini F. Gandini A. (2018). New insights into the runt domain of RUNX2 in melanoma cell proliferation and migration. *Cells* 7:220. 10.3390/cells7110220 30463392 PMC6262450

[B13] Fields J. Dumaop W. Langford T. D. Rockenstein E. Masliah E. (2014). Role of neurotrophic factor alterations in the neurodegenerative process in HIV associated neurocognitive disorders. *J. Neuroimmune Pharmacol.* 9 102–116. 10.1007/s11481-013-9520-2 24510686 PMC3973421

[B14] Fraldi A. Klein A. D. Medina D. L. Settembre C. (2016). Brain disorders due to lysosomal dysfunction. *Annu. Rev. Neurosci.* 39 277–295. 10.1146/annurev-neuro-070815-014031 27090953

[B15] Guillot F. Kemppainen S. Lavasseur G. Miettinen P. O. Laroche S. Tanila H. (2016). Brain-specific basal and novelty-induced alternations in PI3K-Akt and MAPK/ERK signaling in a middle-aged AβPP/PS1 mouse model of Alzheimer’s disease. *J. Alzheimers Dis.* 51 1157–1173. 10.3233/jad-150926 26923018

[B16] Hokama M. Oka S. Leon J. Ninomiya T. Honda H. Sasaki K. (2014). Altered expression of diabetes-related genes in Alzheimer’s disease brains: the Hisayama study. *Cereb. Cortex* 24 2476–2488. 10.1093/cercor/bht101 23595620 PMC4128707

[B17] Huang R. Huang J. Cathcart H. Smith S. Poduslo S. E. (2007). Genetic variants in brain-derived neurotrophic factor associated with Alzheimer’s disease. *J. Med. Genet.* 44:e66. 10.1136/jmg.2006.044883 17293537 PMC2598055

[B18] Huang Y. Mucke L. (2012). Alzheimer mechanisms and therapeutic strategies. *Cell* 148 1204–1222. 10.1016/j.cell.2012.02.040 22424230 PMC3319071

[B19] Jia L. Zhu M. Kong C. Pang Y. Zhang H. Qiu Q. (2020). Blood neuro-exosomal synaptic proteins predict Alzheimer’s disease at the asymptomatic stage. *Alzheimers Dement.* 17 49–60. 10.1002/alz.12166 32776690 PMC7984076

[B20] Jiang S. Tang L. Zhao N. Yang W. Qiu Y. Chen H. Z. A. (2016). Systems view of the differences between APOE ε4 carriers and non-carriers in Alzheimer’s disease. *Front. Aging Neurosci.* 8:171. 10.3389/fnagi.2016.00171 27462267 PMC4941795

[B21] Khan A. Ali A. Junaid M. Liu C. Kaushik A. C. Cho W. C. S. (2018). Identification of novel drug targets for diamond-blackfan anemia based on RPS19 gene mutation using protein-protein interaction network. *BMC Syst. Biol.* 12(Suppl. 4):39. 10.1186/s12918-018-0563-0 29745857 PMC5998885

[B22] Kumar A. Singh A. Ekavali. (2015). A review on Alzheimer’s disease pathophysiology and its management: an update. *Pharmacol. Rep.* 67 195–203. 10.1016/j.pharep.2014.09.004 25712639

[B23] Laske C. Stransky E. Leyhe T. Eschweiler G. W. Wittorf A. Richartz E. (2006). Stage-dependent BDNF serum concentrations in Alzheimer’s disease. *J. Neural Trans.* 113 1217–1224. 10.1007/s00702-005-0397-y 16362629

[B24] Leek J. T. Johnson W. E. Parker H. S. Jaffe A. E. Storey J. D. (2012). The sva package for removing batch effects and other unwanted variation in high-throughput experiments. *Bioinformatics* 28 882–883. 10.1093/bioinformatics/bts034 22257669 PMC3307112

[B25] Lei Q. Y. Zhang H. Zhao B. Zha Z. Y. Bai F. Pei X. H. (2008). TAZ promotes cell proliferation and epithelial-mesenchymal transition and is inhibited by the hippo pathway. *Mol. Cell Biol.* 28 2426–2436. 10.1128/mcb.01874-07 18227151 PMC2268418

[B26] Marmigère F. Givalois L. Rage F. Arancibia S. Tapia-Arancibia L. (2003). Rapid induction of BDNF expression in the hippocampus during immobilization stress challenge in adult rats. *Hippocampus* 13 646–655. 10.1002/hipo.10109 12921353

[B27] McKhann G. M. Knopman D. S. Chertkow H. Hyman B. T. Jack C. R. Jr. Kawas C. H. (2011). The diagnosis of dementia due to Alzheimer’s disease: recommendations from the National Institute on Aging-Alzheimer’s Association workgroups on diagnostic guidelines for Alzheimer’s disease. *Alzheimers Dement.* 7 263–269. 21514250 10.1016/j.jalz.2011.03.005 PMC3312024

[B28] Pineda S. Real F. X. Kogevinas M. Carrato A. Chanock S. J. Malats N. (2015). Integration analysis of three omics data using penalized regression methods: an application to bladder cancer. *PLoS Genet.* 11:e1005689. 10.1371/journal.pgen.1005689 26646822 PMC4672920

[B29] Prince M. Bryce R. Albanese E. Wimo A. Ribeiro W. Ferri C. P. (2013). The global prevalence of dementia: a systematic review and metaanalysis. *Alzheimers Dement.* 9 63–75.e2. 23305823 10.1016/j.jalz.2012.11.007

[B30] Querfurth H. W. LaFerla F. M. (2010). Alzheimer’s disease. *N. Engl. J. Med.* 362 329–344. 10.1056/NEJMra0909142 20107219

[B31] Ren Z. Yu J. Wu Z. Si W. Li X. Liu Y. (2018). MicroRNA-210-5p contributes to cognitive impairment in early vascular dementia rat model through targeting snap25. *Front. Mol. Neurosci.* 11:388. 10.3389/fnmol.2018.00388 30483048 PMC6243094

[B32] Ritchie M. E. Phipson B. Wu D. Hu Y. Law C. W. Shi W. (2015). limma powers differential expression analyses for RNA-sequencing and microarray studies. *Nucleic Acids Res* 43:e47. 10.1093/nar/gkv007 25605792 PMC4402510

[B33] Robin X. Turck N. Hainard A. Tiberti N. Lisacek F. Sanchez J. C. (2011). pROC: an open-source package for R and S+ to analyze and compare ROC curves. *BMC Bioinform.* 12:77. 10.1186/1471-2105-12-77 21414208 PMC3068975

[B34] Rogaev E. I. Sherrington R. Rogaeva E. A. Levesque G. Ikeda M. Liang Y. (1995). Familial Alzheimer’s disease in kindreds with missense mutations in a gene on chromosome 1 related to the Alzheimer’s disease type 3 gene. *Nature* 376 775–778. 10.1038/376775a0 7651536

[B35] Salter M. W. Stevens B. (2017). Microglia emerge as central players in brain disease. *Nat. Med.* 23 1018–1027. 10.1038/nm.4397 28886007

[B36] Sayer R. Law E. Connelly P. J. Breen K. C. (2004). Association of a salivary acetylcholinesterase with Alzheimer’s disease and response to cholinesterase inhibitors. *Clin. Biochem.* 37 98–104. 10.1016/j.clinbiochem.2003.10.007 14725939

[B37] Sherrington R. Rogaev E. I. Liang Y. Rogaeva E. A. Levesque G. Ikeda M. (1995). Cloning of a gene bearing missense mutations in early-onset familial Alzheimer’s disease. *Nature* 375 754–760. 7596406 10.1038/375754a0

[B38] Solarski M. Wang H. Wille H. Schmitt-Ulms G. (2018). Somatostatin in Alzheimer’s disease: a new role for an old player. *Prion* 12 1–8. 10.1080/19336896.2017.1405207 29192843 PMC5871028

[B39] Sorbi S. Forleo P. Tedde A. Cellini E. Ciantelli M. Bagnoli S. (2001). Genetic risk factors in familial Alzheimer’s disease. *Mech. Ageing Dev.* 122 1951–1960. 11589913 10.1016/s0047-6374(01)00308-6

[B40] Stopa E. G. Tanis K. Q. Miller M. C. Nikonova E. V. Podtelezhnikov A. A. Finney E. M. (2018). Comparative transcriptomics of choroid plexus in Alzheimer’s disease, frontotemporal dementia and Huntington’s disease: implications for CSF homeostasis. *Fluids Barriers CNS* 15:18. 10.1186/s12987-018-0102-9 PMC5977762 29848382

[B41] Subramanian A. Tamayo P. Mootha V. K. Mukherjee S. Ebert B. L. Gillette M. A. (2005). Gene set enrichment analysis: a knowledge-based approach for interpreting genome-wide expression profiles. *Proc. Natl. Acad. Sci. U.S.A.* 102 15545–15550. 10.1073/pnas.0506580102 16199517 PMC1239896

[B42] Tanzi R. E. Bertram L. (2005). Twenty years of the Alzheimer’s disease amyloid hypothesis: a genetic perspective. *Cell* 120 545–555. 10.1016/j.cell.2005.02.008 15734686

[B43] Troyanskaya O. Cantor M. Sherlock G. Brown P. Hastie T. Tibshirani R. (2001). Missing value estimation methods for DNA microarrays. *Bioinformatics* 17 520–525. 10.1093/bioinformatics/17.6.520 11395428

[B44] Wang E. Zhu H. Wang X. Gower A. C. Wallack M. Blusztajn J. K. (2017). Amylin treatment reduces neuroinflammation and ameliorates abnormal patterns of gene expression in the cerebral cortex of an Alzheimer’s disease mouse model. *J. Alzheimers Dis.* 56 47–61. 10.3233/jad-160677 27911303 PMC5331853

[B45] Wang S. P. Wang L. H. (2016). Disease implication of hyper-Hippo signalling. *Open Biol.* 6:160119. 10.1098/rsob.160119 27805903 PMC5090056

[B46] Wimo A. Guerchet M. Ali G. C. Wu Y. T. Prina A. M. Winblad B. (2017). The worldwide costs of dementia 2015 and comparisons with 2010. *Alzheimers Dement.* 13 1–7. 10.1016/j.jalz.2016.07.150 27583652 PMC5232417

[B47] Wingo T. S. Lah J. J. Levey A. I. Cutler D. J. (2012). Autosomal recessive causes likely in early-onset Alzheimer disease. *Arch. Neurol.* 69 59–64. 10.1001/archneurol.2011.221 21911656 PMC3332307

[B48] Yao F. Zhang K. Zhang Y. Guo Y. Li A. Xiao S. (2018). Identification of blood biomarkers for Alzheimer’s disease through computational prediction and experimental validation. *Front. Neurol.* 9:1158. 10.3389/fneur.2018.01158 30671019 PMC6331438

[B49] Zetterberg H. Andreasen N. Blennow K. (2004). Increased cerebrospinal fluid levels of transforming growth factor-beta1 in Alzheimer’s disease. *Neurosci. Lett.* 367 194–196. 10.1016/j.neulet.2004.06.001 15331151

[B50] Zhou M. Haque R. U. Dammer E. B. Duong D. M. Ping L. Johnson E. C. B. (2020). Targeted mass spectrometry to quantify brain-derived cerebrospinal fluid biomarkers in Alzheimer’s disease. *Clin. Proteom.* 17:19. 10.1186/s12014-020-09285-8 PMC7257173 32514259

[B51] Zhu M. Jia L. Li F. Jia J. (2020). Identification of KIAA0513 and other hub genes associated with Alzheimer disease using weighted gene coexpression network analysis. *Front. Genet.* 11:981. 10.3389/fgene.2020.00981 33005179 PMC7483929

[B52] Zhu X. Lee H. G. Raina A. K. Perry G. Smith M. A. (2002). The role of mitogen-activated protein kinase pathways in Alzheimer’s disease. *Neurosignals* 11 270–281. 12566928 10.1159/000067426

[B53] Zhu X. Rottkamp C. A. Raina A. K. Brewer G. J. Ghanbari H. A. Boux H. (2000). Neuronal CDK7 in hippocampus is related to aging and Alzheimer disease. *Neurobiol. Aging* 21 807–813. 10.1016/s0197-4580(00)00217-7 11124424

[B54] Zhu X. Zhan J. Wang J. Li M. (2019). Changes and significance of SYP and GAP-43 expression in the hippocampus of CIH rats. *Int. J. Med. Sci.* 16 394–402. 10.7150/ijms.28359 30911273 PMC6428973

[B55] Zipser B. D. Johanson C. E. Gonzalez L. Berzin T. M. Tavares R. Hulette C. M. (2007). Microvascular injury and blood-brain barrier leakage in Alzheimer’s disease. *Neurobiol. Aging* 28 977–986. 16782234 10.1016/j.neurobiolaging.2006.05.016

[B56] Zou Y. He W. Wang K. Han H. Xiao T. Chen X. (2018). Identification of rare RTN3 variants in Alzheimer’s disease in Han Chinese. *Hum. Genet.* 137 141–150. 10.1007/s00439-018-1868-1 29356939

