# Supplementary material for: Exploring the Key Genes and Identification of Potential Diagnosis Biomarkers in Alzheimer’s Disease Using Bioinformatics Analysis
Source: Front Aging Neurosci. 2021 Jun 14;13:602781. doi: 10.3389/fnagi.2021.602781 (PMC8236887; doi:10.3389/fnagi.2021.602781)
Supplement: Supplementary file 1 [file Data_Sheet_1.PDF]

Supplementary information

Exploring the Key Genes and Identification of Potential diagnosis Biomarkers in the human brain of Alzheimer's disease Using Bioinformatics Analysis

Wuhan Yu[1], Weihua Yu[2], Yan Yang[3], Yang Lü[1]*

Department of Geriatrics, The First Affiliated Hospital of Chongqing Medical University, Chongqing 400016, China

corresponding author: Prof. Yang Lü, Department of Geriatrics, The First Affiliated Hospital of Chongqing Medical University, Chongqing 400016, China, Add: No.1 Youyi Road, Yuzhong District, Chongqing 400016, China, Tel: +86-23-89011622; Fax: +86-23-68811487; E-mail: lyu_yang@126.com

Figure legend

Supplementary Figure 1. Heatmap of common differentially expressed genes.

Supplementary Figure 2. Results from the principal component analysis for consensus DEGs of different brain regions. Abbreviations: AD, Alzheimer's disease; ND. Non-demented.

Supplementary Figure 3. Receiver operating characteristic (ROC) curves analysis of top ten hub genes in combined datasets. (A) ROC curves of top 1-5 hub genes. (B) ROC curves of top 5-10 hub genes. AUC: Area Under the Curve.

Supplementary Figure 4. Gene Set Enrichment Analysis (GSEA) of the rest of top ten hub genes based on GSE48350. (A) SMAD4. (B) SLC32A1. (C) YAP1. (D) SYP. (E) SNAP25. (F) VDAC1. (G) ATP5B. (H) ITPKB.
