# Supplementary figures and images for: Exploring the Key Genes and Identification of Potential Diagnosis Biomarkers in Alzheimer’s Disease Using Bioinformatics Analysis
Source: Front Aging Neurosci. 2021 Jun 14;13:602781. doi: 10.3389/fnagi.2021.602781 (PMC8236887; doi:10.3389/fnagi.2021.602781)

GSE33000

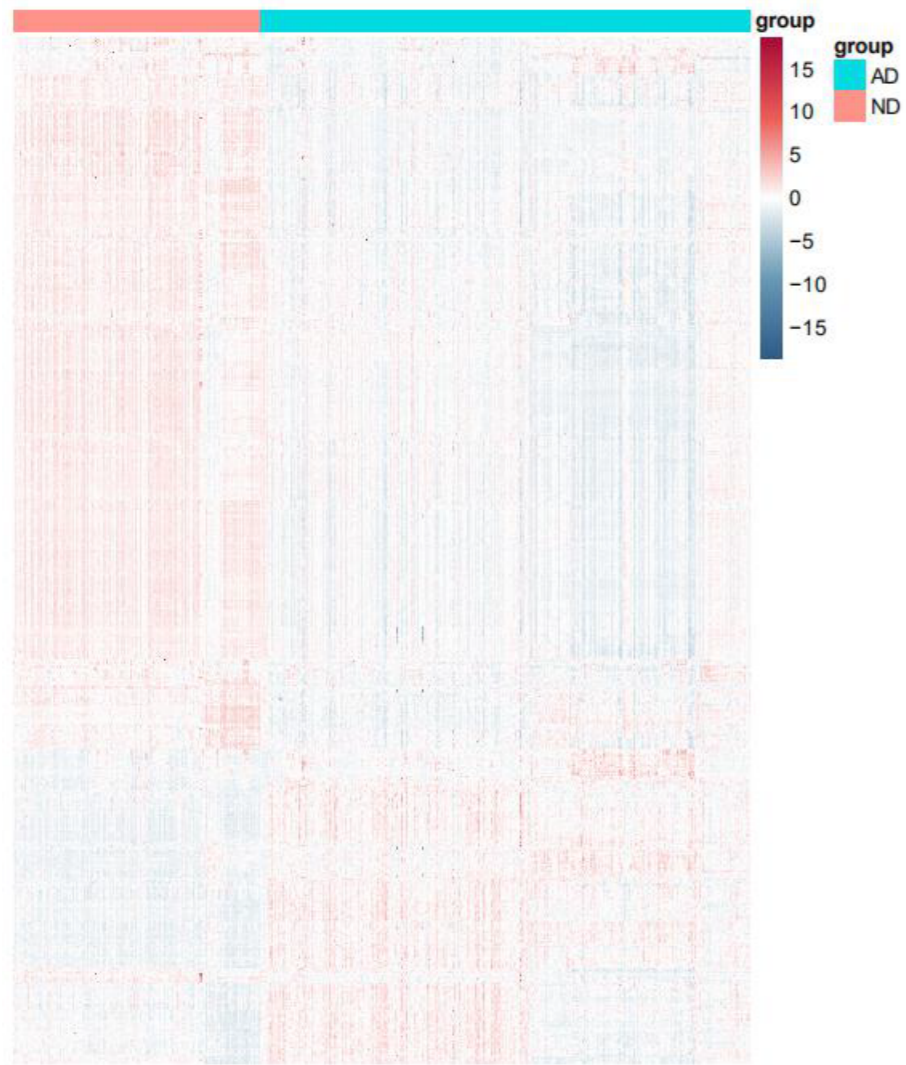

GSE36980

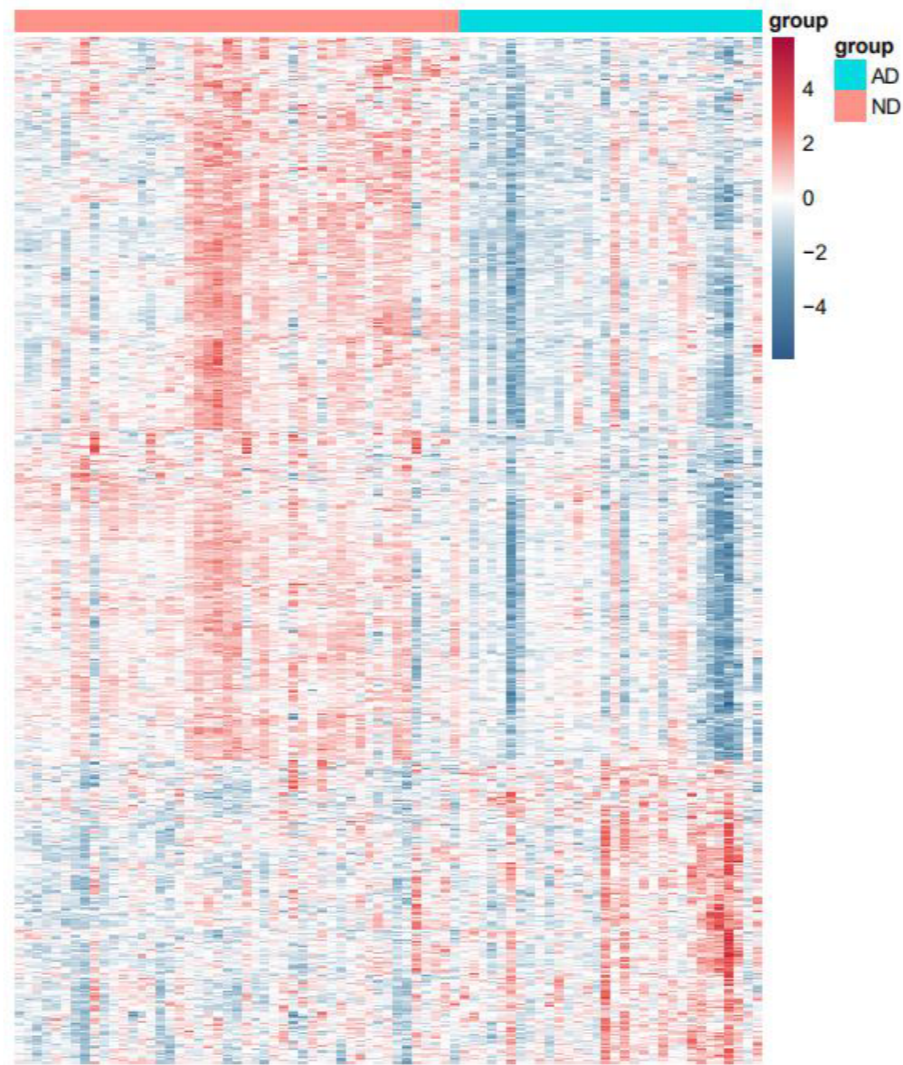

GSE122063

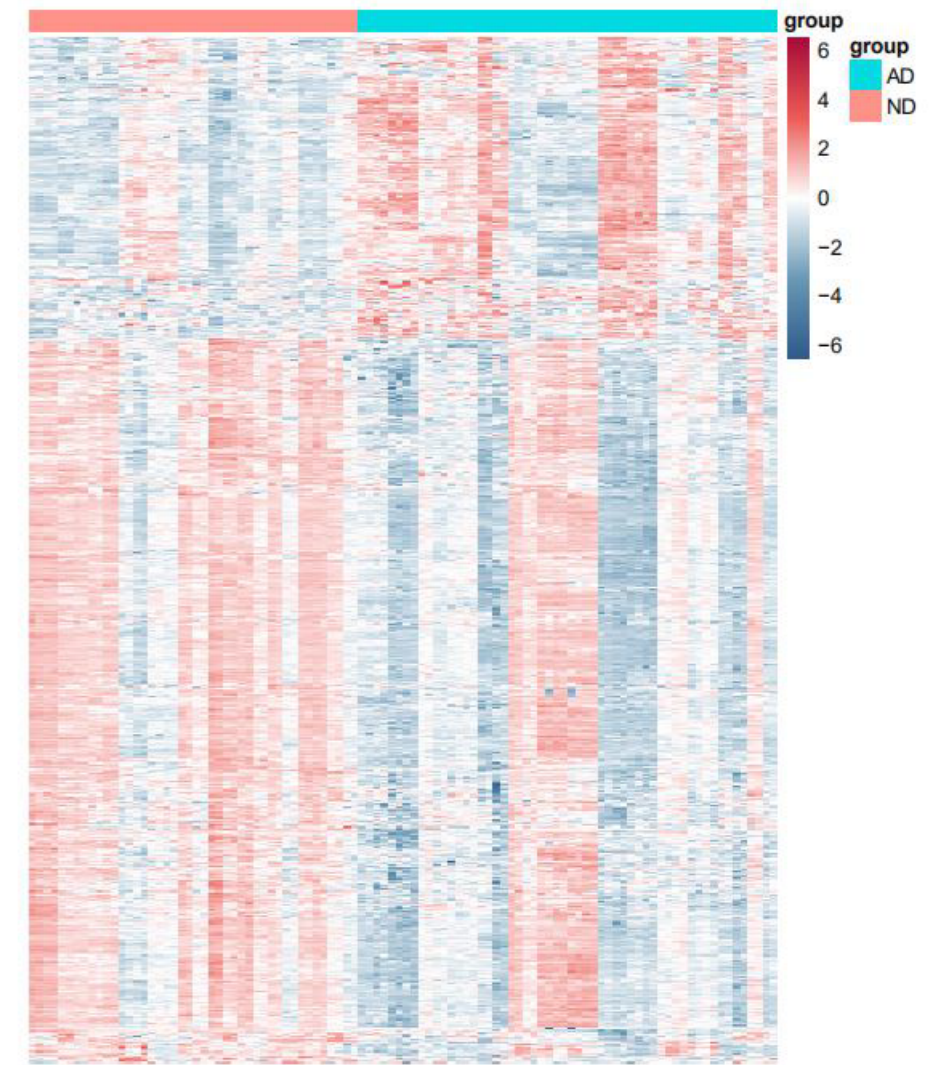

GSE5281

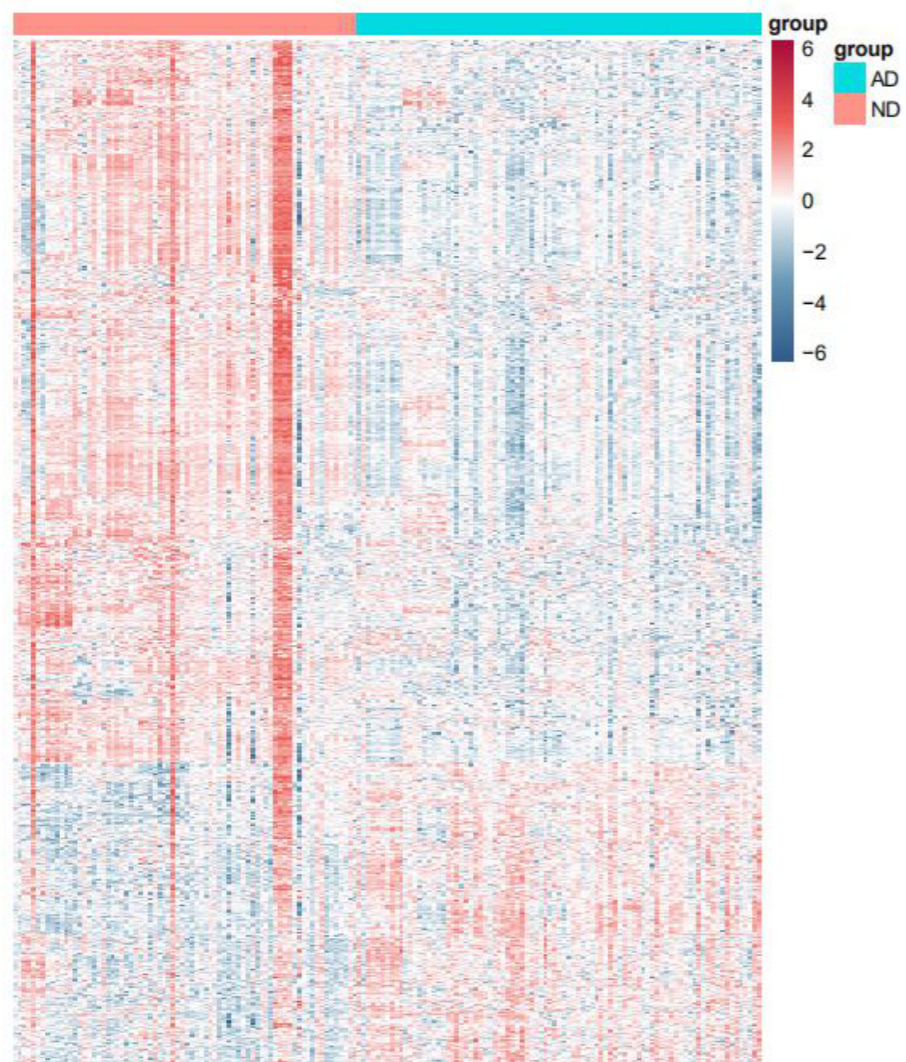

GSE48350

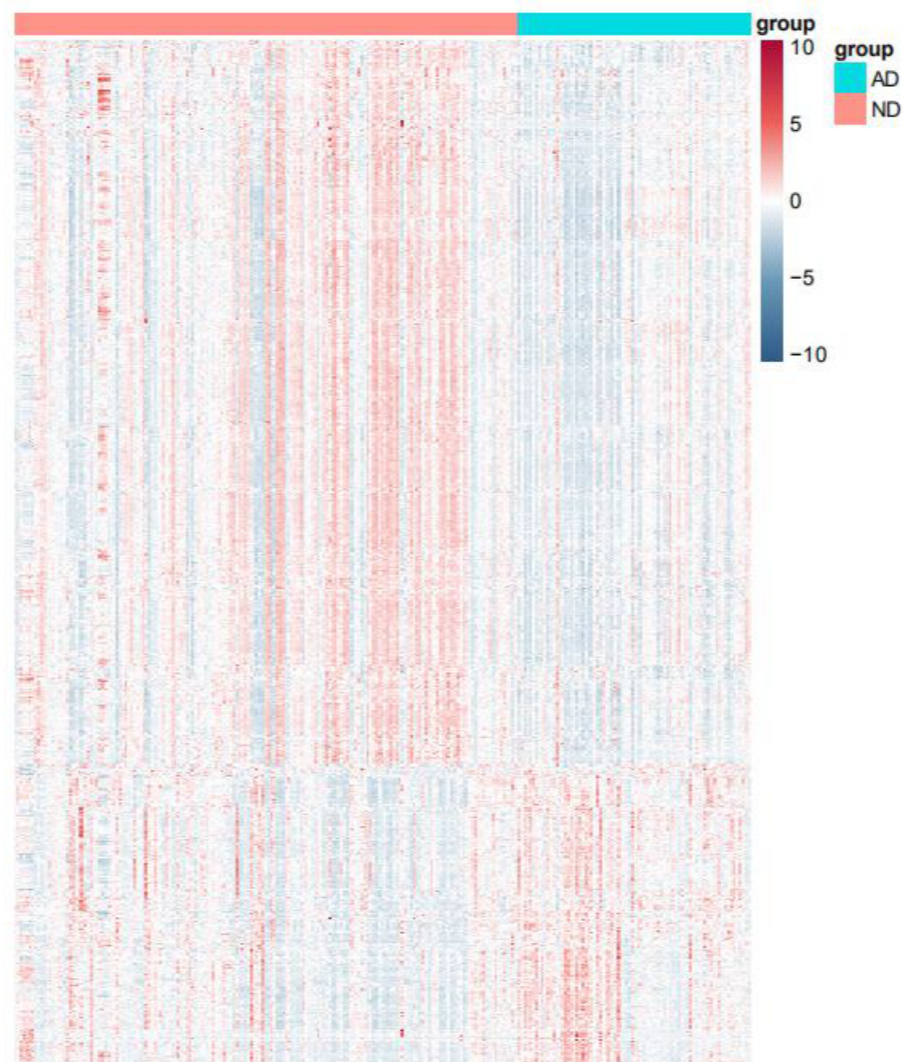

Supplement: Supplementary file 2 [file Data_Sheet_2.PDF]

PCA2

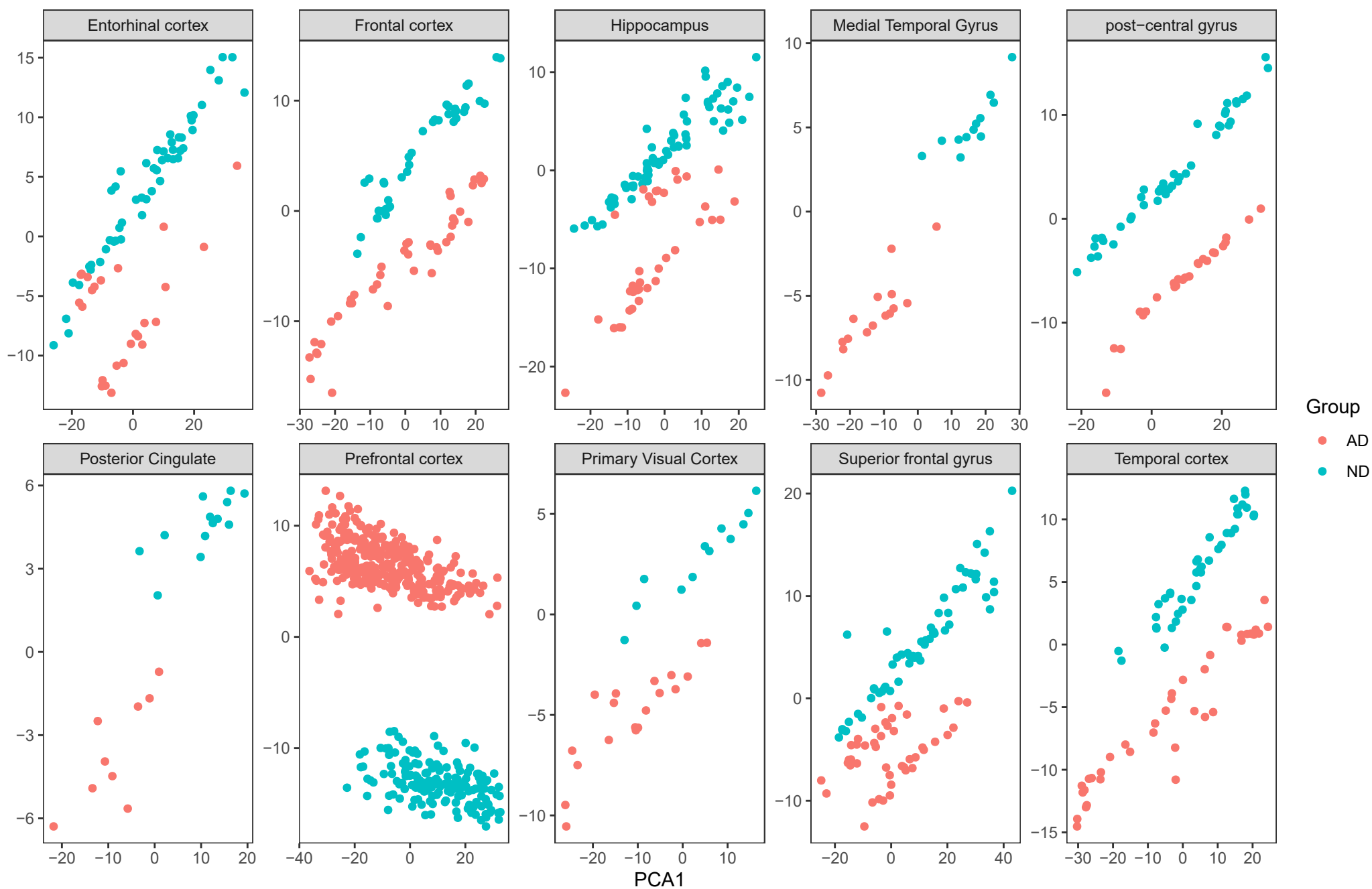

Group

● AD  
● ND

PCA1

Supplement: Supplementary file 3 [file Data_Sheet_3.PDF]

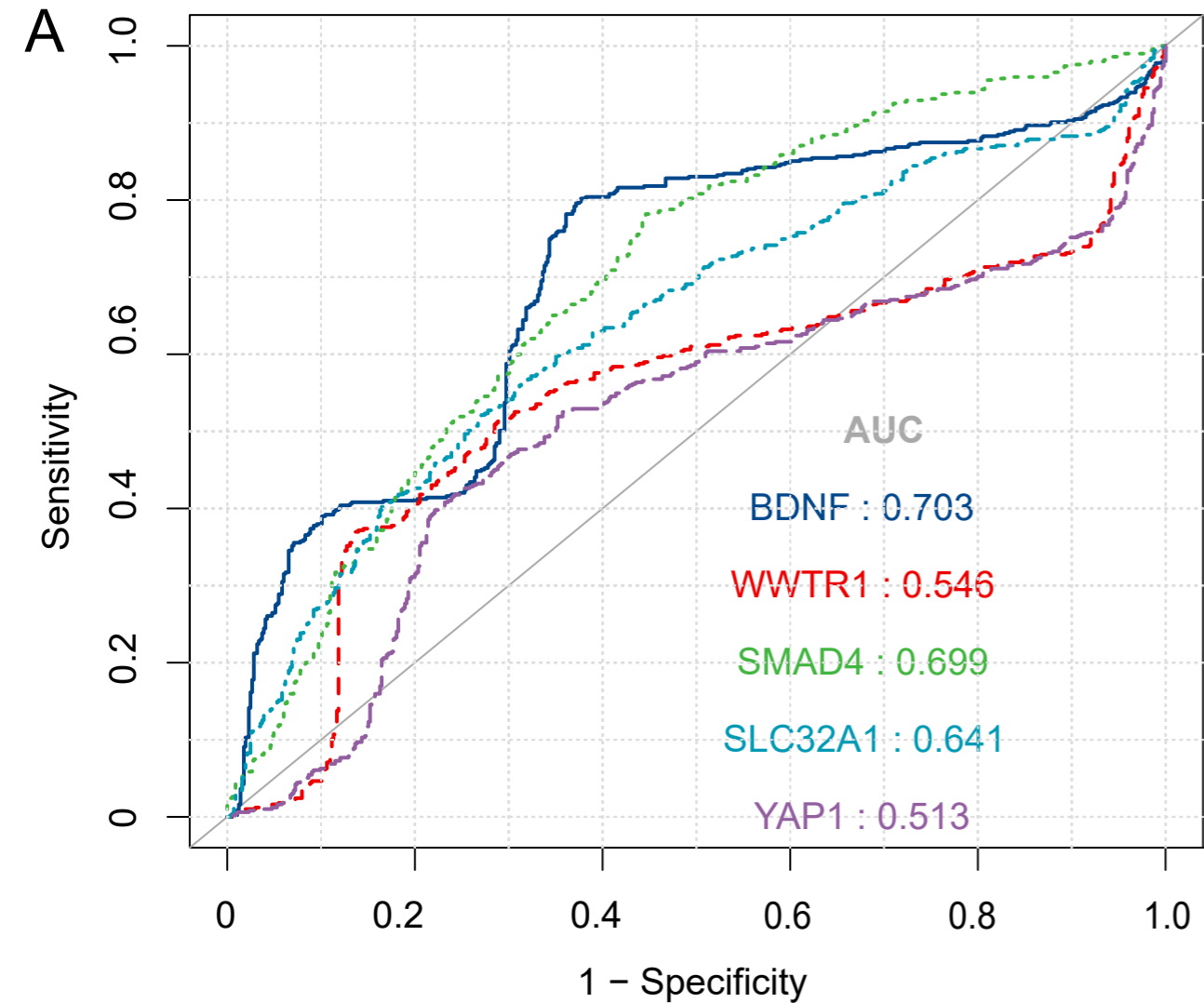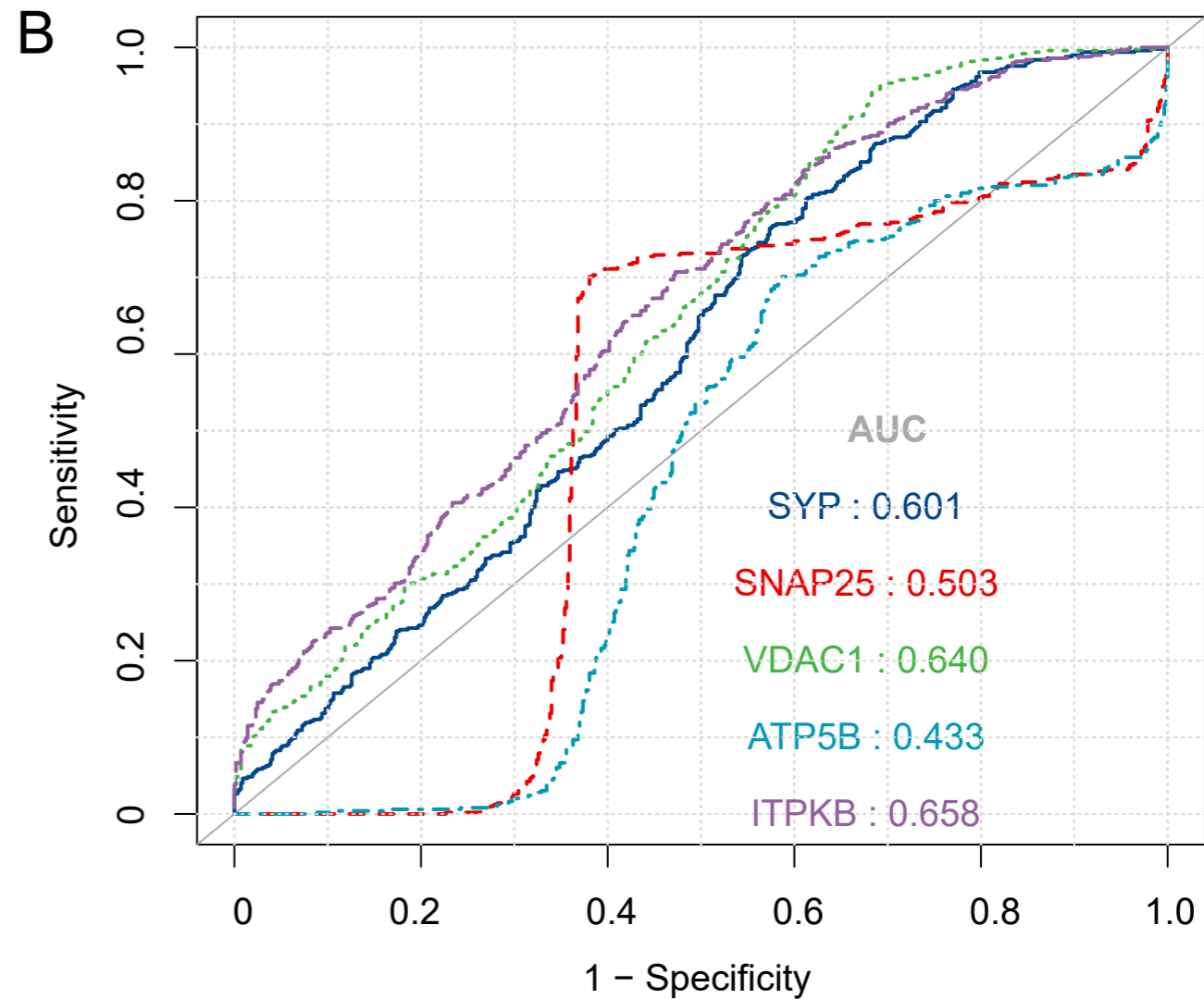

Supplement: Supplementary file 4 [file Data_Sheet_4.PDF]

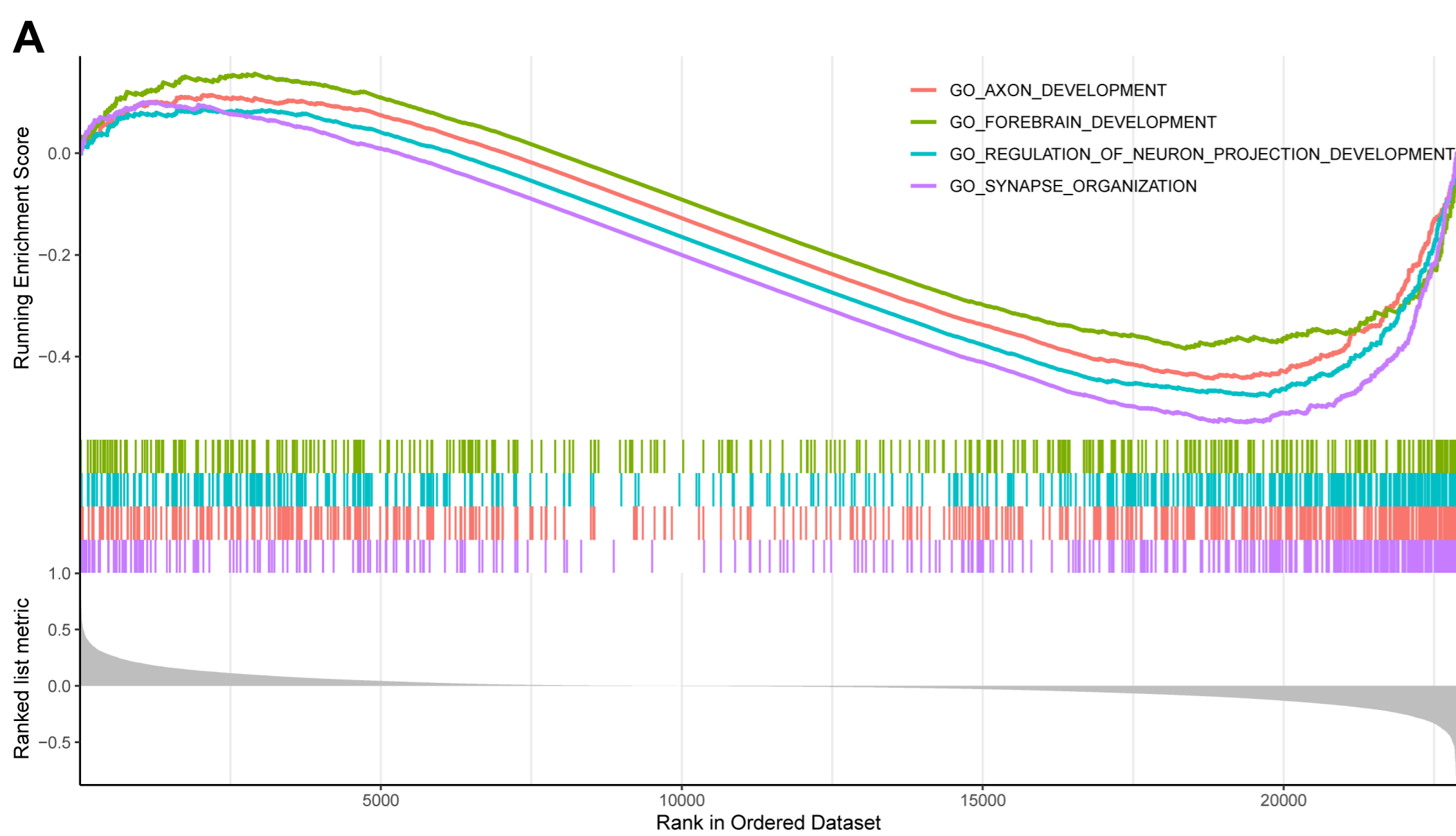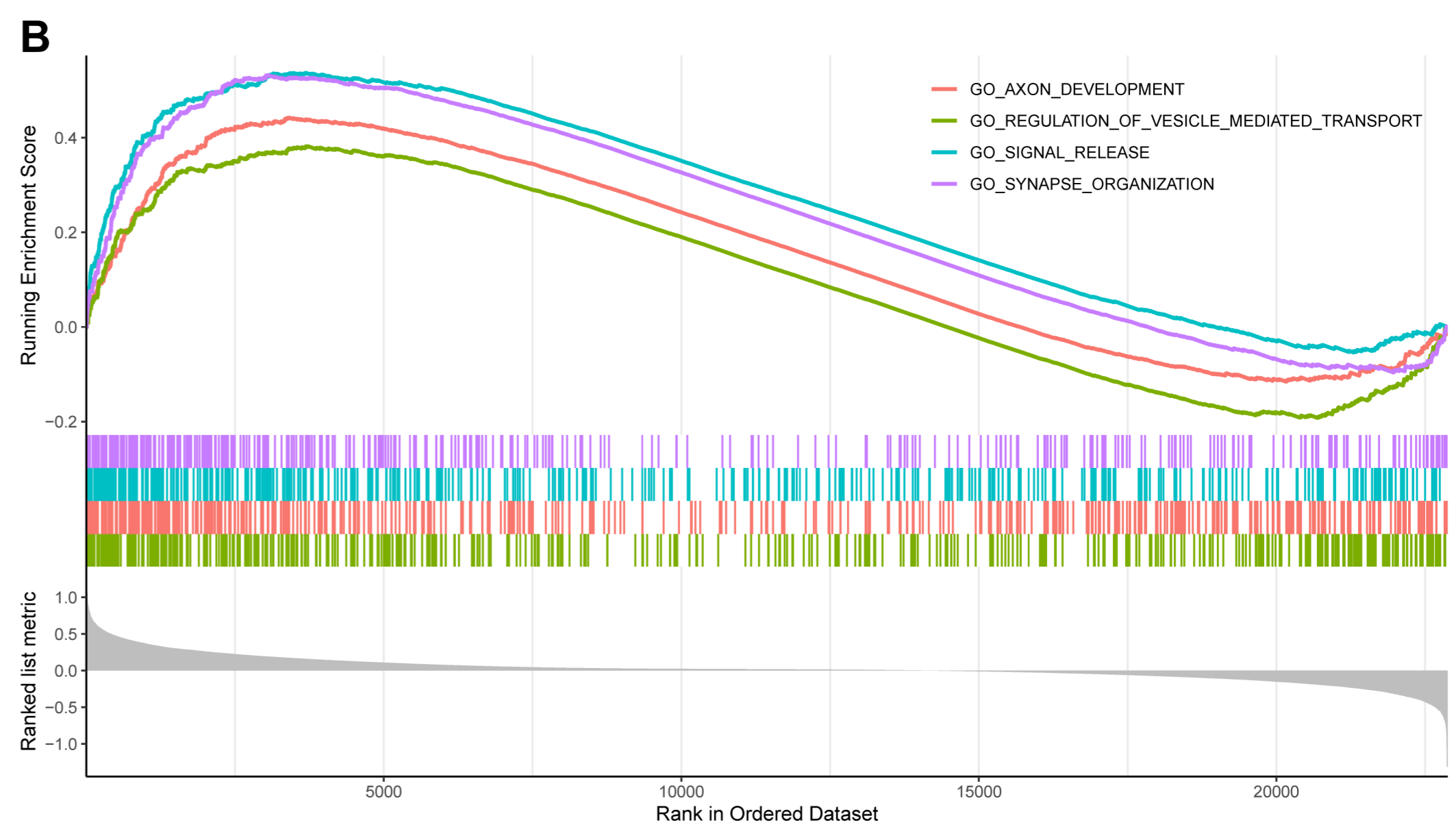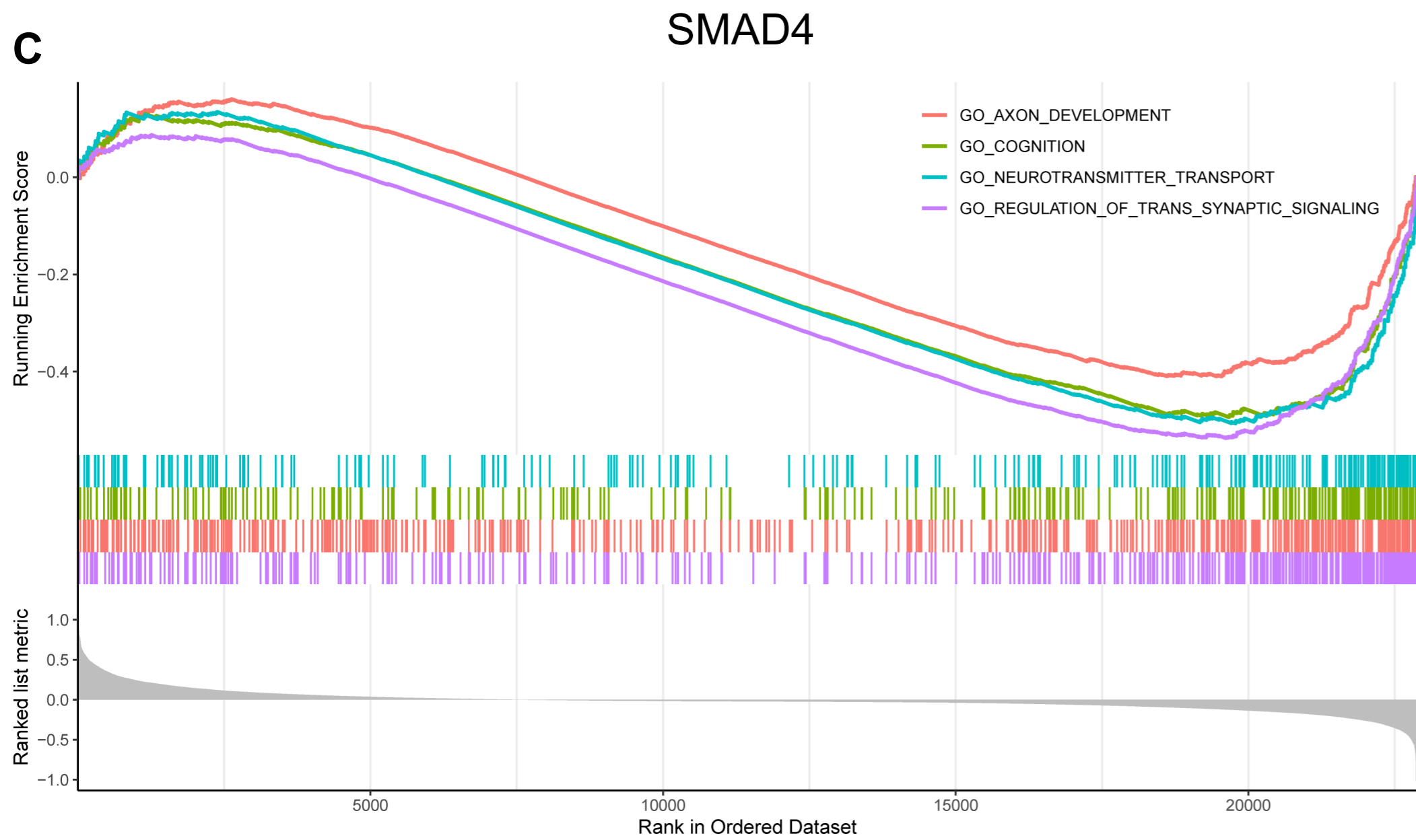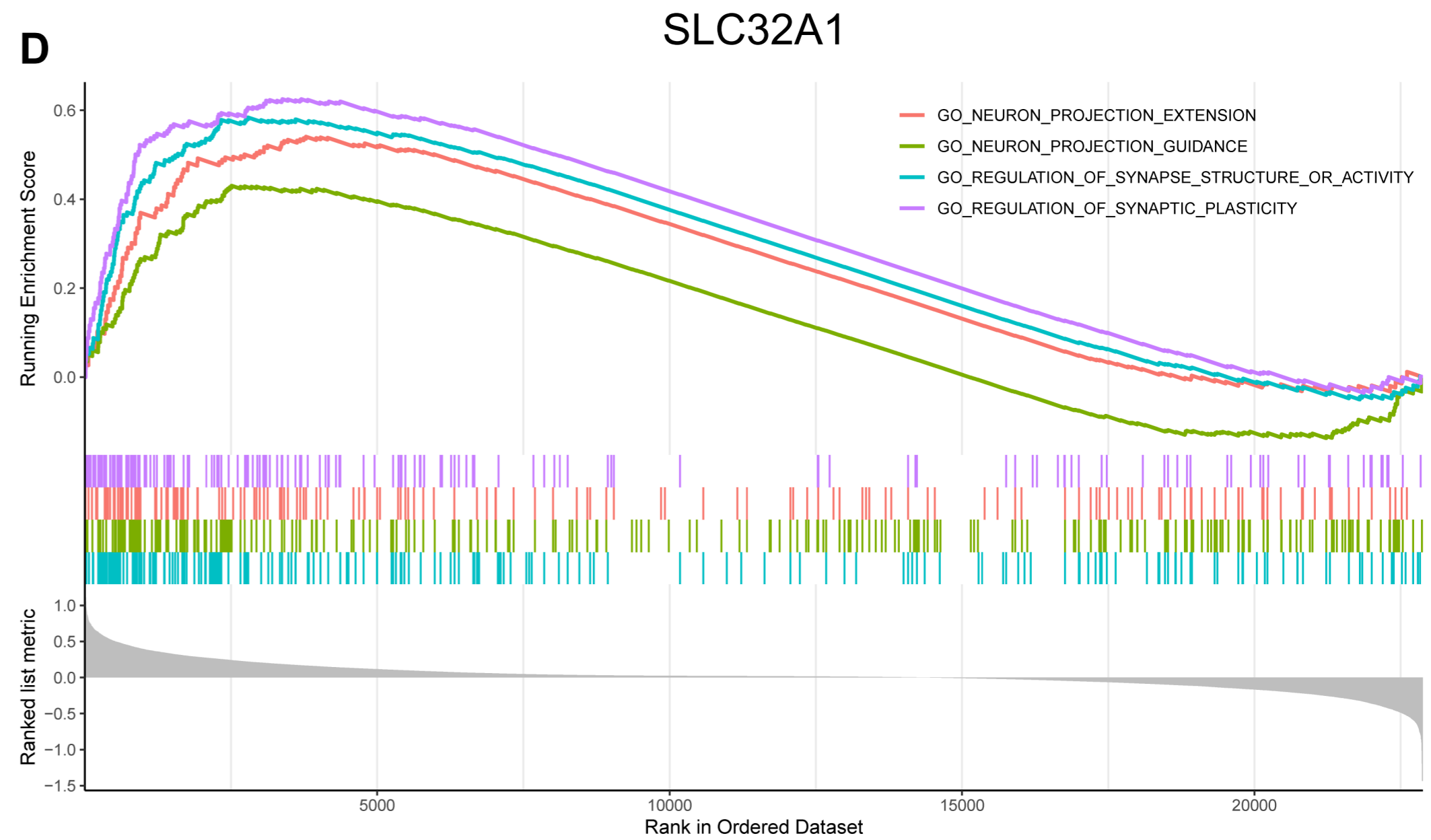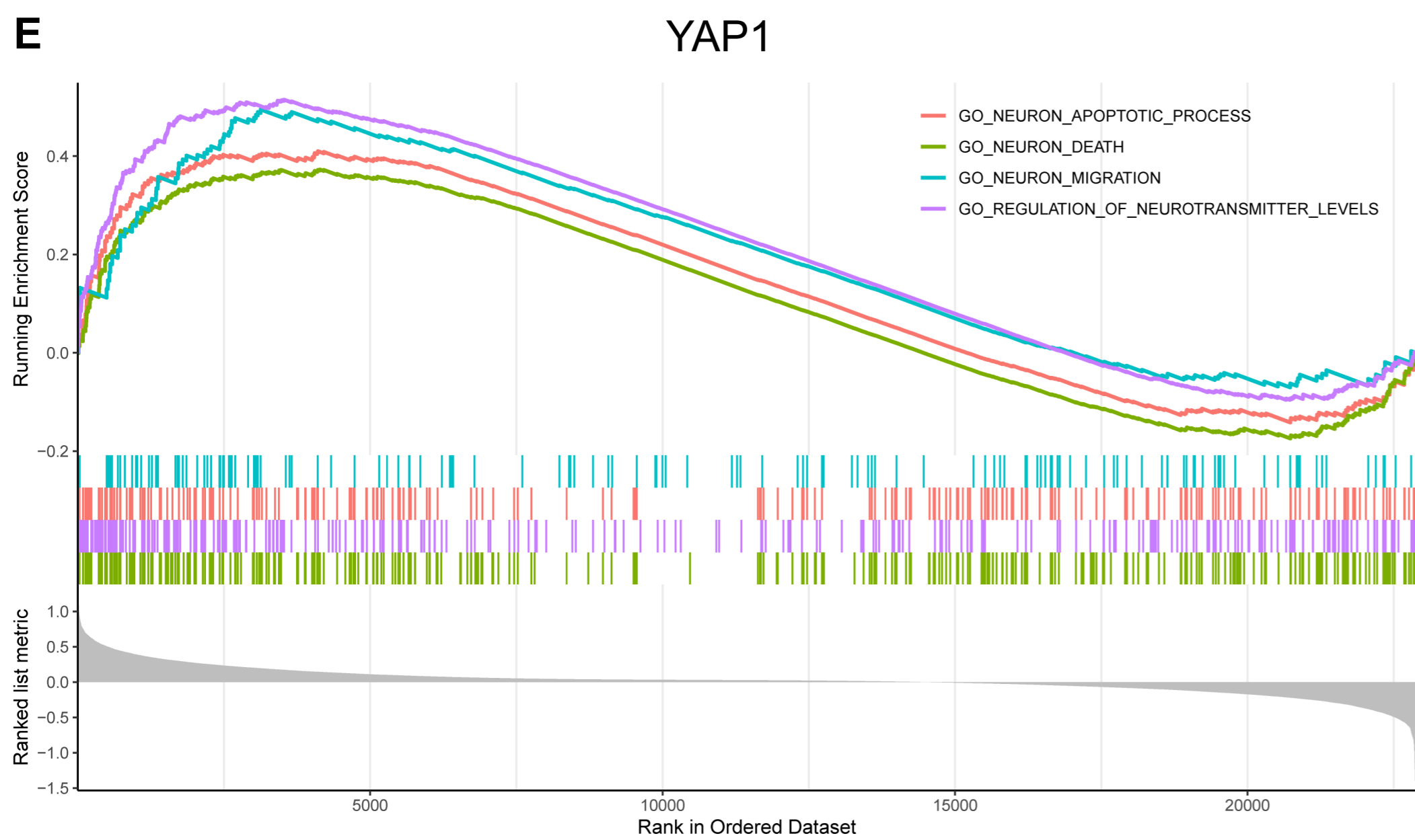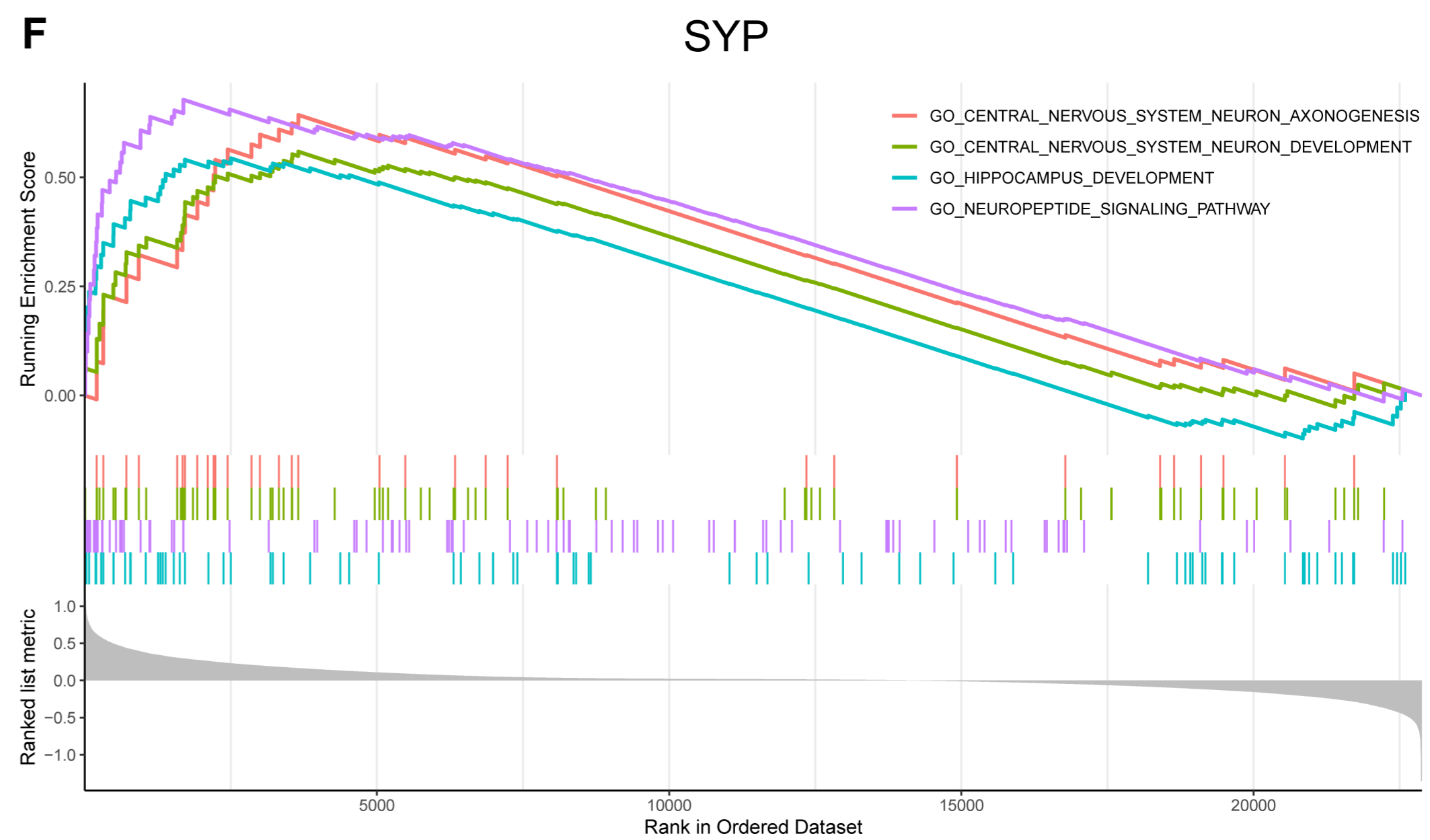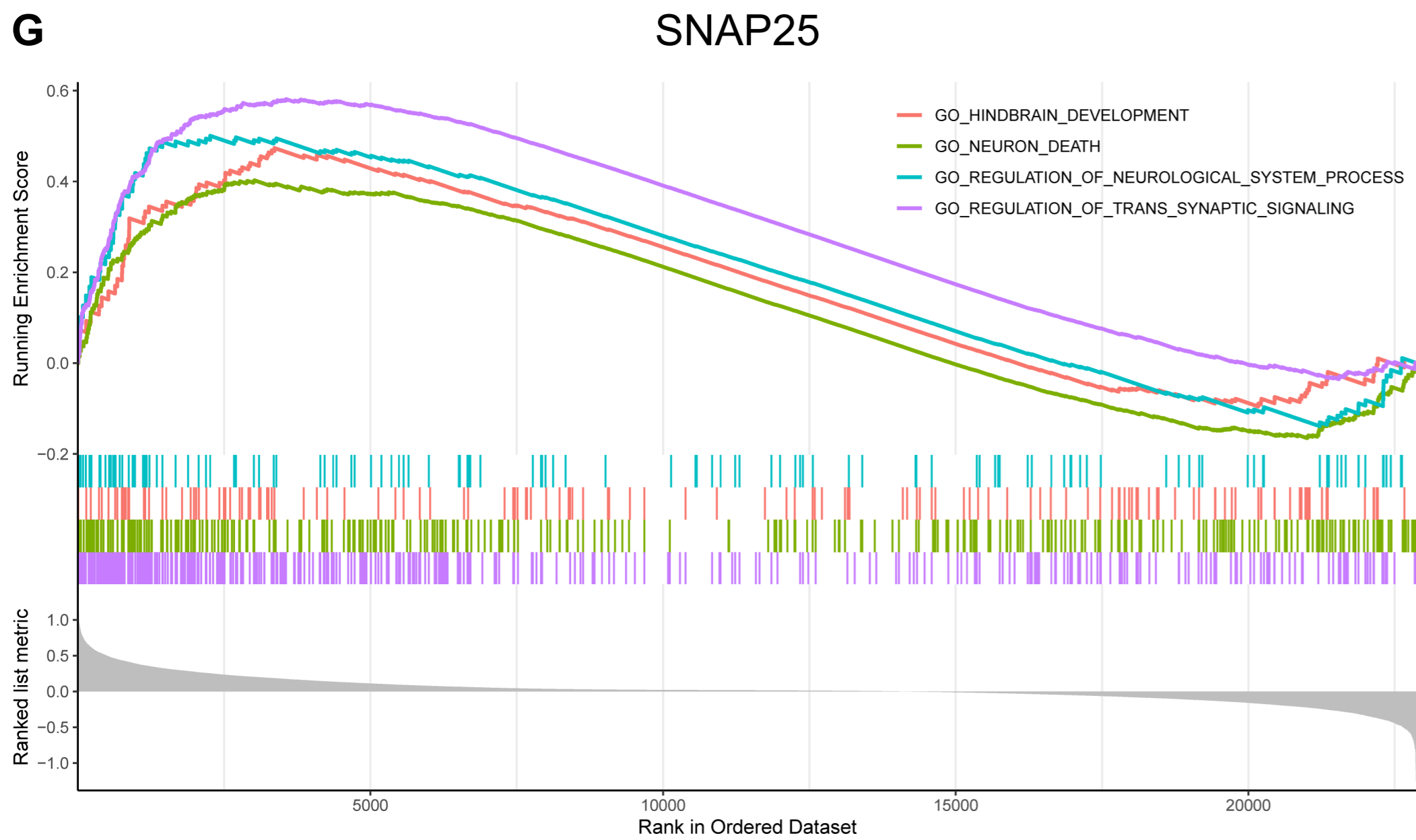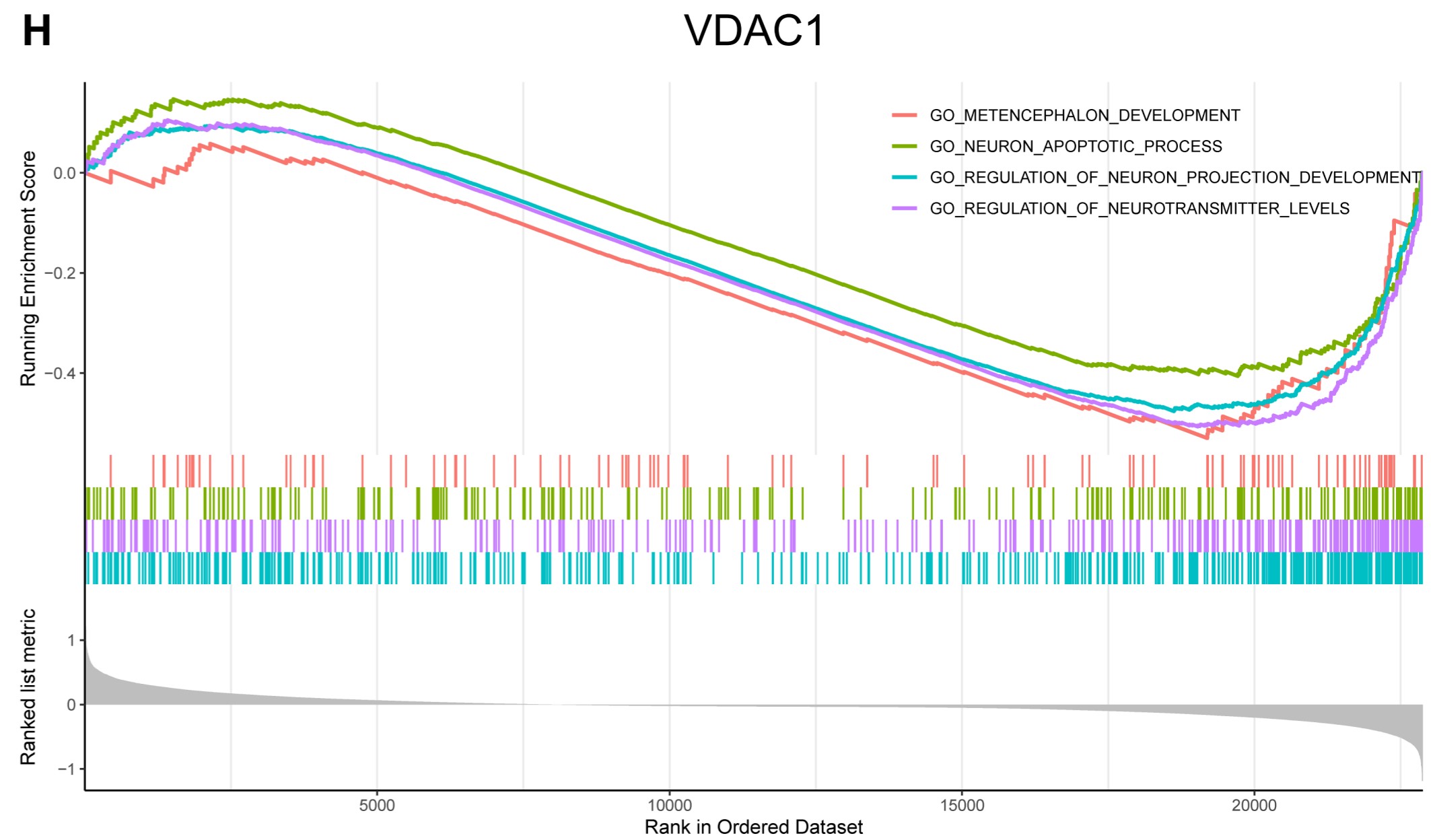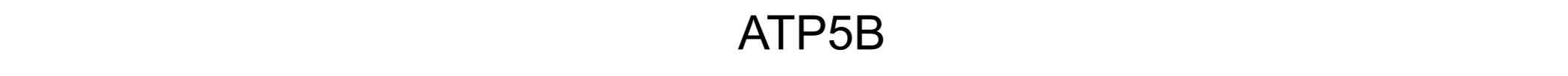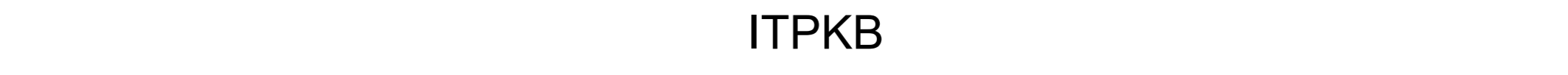

Supplement: Supplementary file 5 [file Data_Sheet_5.PDF]
